# Design, Synthesis and SAR Study of Novel Trisubstituted Pyrimidine Amide Derivatives as CCR4 Antagonists

**DOI:** 10.3390/molecules19033539

**Published:** 2014-03-21

**Authors:** Libao Xu, Yang Zhang, Wenjie Dai, Ying Wang, Dan Jiang, Lili Wang, Junhai Xiao, Xiaohong Yang, Song Li

**Affiliations:** 1 School of Pharmaceutical Sciences, Jilin University, Changchun 130021, China; E-Mails: xulibao2003@163.com (L.X.); lis@bmi.ac.cn (S.L.); 2 Laboratory of Computer-Aided Drug Design & Discovery, Beijing Institute of Pharmacology & Toxicology, Beijing 100850, China; E-Mails: jiangdan@263.net.cn (D.J.); wangll63@126.com (L.W.); 3 Department of Immunology, School of Basic Medical Sciences, Key Laboratory of Medical Immunology, Ministry of Health, Peking University Health Science Center, Beijing 100191, China; E-Mails: byzhangyang08@163.com (Y.Z.); yw@bjmu.edu.cn (Y.W.); 4 Shenyang Pharmaceutical University, 103 Wenhua Road, Shenhe District, Shenyang 110016, China; E-Mail: wenjiedai.2009@163.com

**Keywords:** antagonists, pyrimidine amides, synthesis, structure-activity relationship

## Abstract

The design, synthesis and structure-activity relationship studies of some novel trisubstituted pyrimidine amide derivatives prepared as CCR4 antagonists are described. The activities of these compounds were evaluated by the CCR4-MDC chemotaxis inhibition assay. Compound **1**, which we have previously reported as a potent antagonist of CCR4, was employed as the positive control. The results indicated that most of the synthesized compounds exhibited some chemotaxis inhibition activity against CCR4. Of these new compounds, compounds **6c**, **12a** and **12b**, with IC_50 _values of 0.064, 0.077 and 0.069 μM, respectively, showed higher or similar activity compared with compound **1** (IC_50_ of 0.078 μM). These compounds provide a basis for further structural modifications. The systematic structure-activity relationship of these trisubstituted pyrimidine amide derivatives was discussed based on the obtained experimental data. The results from the SAR study may be useful for identifying more potent CCR4 antagonists.

## 1. Introduction

Chemokines are a large family of small chemotactic proteins of 8–10 kDa in size. In humans, chemokine family members now number more than 50. According to the number and relative position of conserved cysteine residues near the N-terminus of each protein, chemokines are divided into four major groups: C, CC, CXC and CX3C. Chemokine receptors, containing a characteristic seven transmembrane structure, belong to the class A family of G protein-coupled receptors (GPCR) and are coupled with the Gαi class of heterotrimeric G proteins. They are also classified into four groups in relation to the subfamily of major chemokine ligands to which they bind. To date, 18 typical chemokine receptors have been described in humans, including one C, ten CC, six CXC and one CX3C chemokine receptor [1,2,3]. Chemokines and their receptors play a key role in immune responses by directing and controlling the migration, activation, differentiation and survival of leukocytes. Binding of chemokines to their innate receptors on the cell surface can trigger a serial complex signaling cascade that leads to inflammatory mediator release, changes in cell morphology, cell proliferation and migration [4,5]. They have been associated with the pathogenesis of multiple inflammatory diseases and cancer progression.

The CC-chemokine receptor 4 (CCR4) belongs to the CC family of chemokine receptors. CCR4 was first cloned from a human basophilic cell line in 1995. Three natural chemokine ligands for CCR4 have been identified as thymus and activation-regulated chemokine (TARC, CCL17), macrophage-derived chemokine (MDC, CCL22) and chemokine-like factor 1 (CKLF1). Among them, TARC and MDC are highly specific biological ligands with strong affinities for CCR4 [6]. CCR4 is mainly expressed on T helper 2 (Th2) type CD4+ T-cells that produce Th2 cytokines, such as interleukin-4, -5 and -13. Th2 cytokines are believed to help accelerate the allergic inflammation response [7]. Upon activation, CCR4 can drive CD4+ Th2 cells to migrate toward inflamed tissues. Up regulation of TARC and MDC as well as accumulation of CCR4-positive cells have been observed in inflamed tissues of patients suffering from allergic diseases. It has been demonstrated that antagonism of MDC or TARC can reduce the migration of T-cells into sites of inflammation [8,9]. Thus CCR4 and its ligands play a critical role in the development of allergic inflammatory conditions such as asthma, atopic dermatitis, and rhinitis [10]. Targeting CCR4 has become a novel therapeutic strategy for these diseases where CCR4 has a central role in pathogenesis.

**Figure 1:**
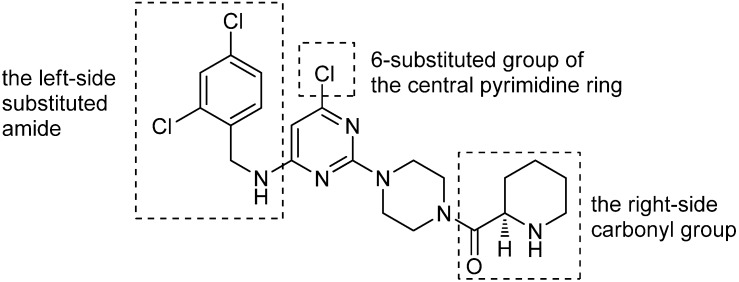
Structure of the compound **1** and the initial strategy for exploring the SAR.

A number of small molecular CCR4 antagonists have been reported in the literature [11,12,13,14]. These highly active antagonists can be divided into three categories based on their skeletal structures: pyrimidine analogs, pyrazine arylsulfonamides and indazole arylsulfonamides. Recently, we reported a novel trisubstituted pyrimidine compound **1** (Figure 1) as a potent antagonist of CCR4 [15]. It can inhibit the migration of CCR4-positive cells induced by MDC, TARC, and CKLF1. We report herein an expanded structure-activity relationship study of compound 1. A series of novel trisubstituted pyrimidine amide analogs have been designed, synthesized and evaluated by a chemotaxis inhibition assay.

## 2. Results and Discussion

### 2.1. Chemistry

**Scheme 1:**
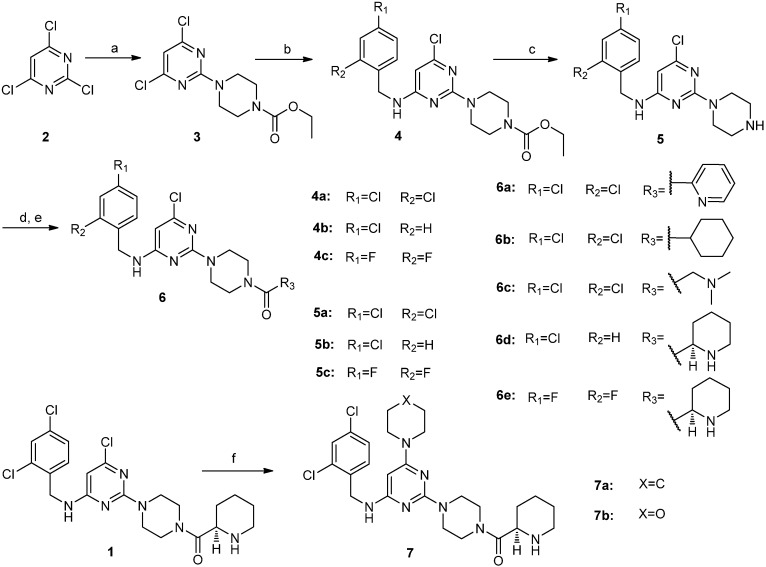
The synthetic routes to compounds **6a**–**e**, **7a** and **7b**.

**Scheme 2:**
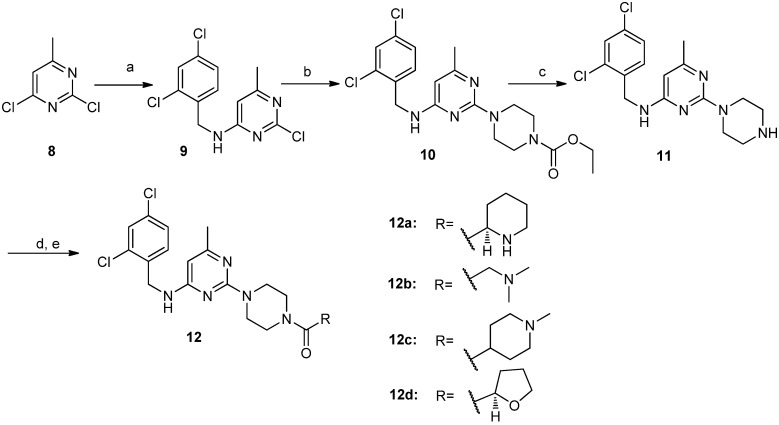
The synthetic route to compounds **12a**–**d**.

In order to explore the initial SAR, we focused on the three major areas of the lead compound **1**, as shown in Figure 1: the right-side carbonyl group, the left-side substituted amide and the 6-substituted group of the central pyrimidine ring. Through optimization of the substituents in these three areas, we sought to identify more potent CCR4 antagonists. Based on these starting points, a series of novel trisubstituted pyrimidine amide compounds **6a**–**e**, **7a**, **7b** and **12a**–**d** were synthesized as summarized in Scheme 1 and Scheme 2 [15,16,17,18]. The synthetic route outlined in Scheme 1 started with reaction of commercially available 2,4,6-trichloropyrimidine (**2**) with N-ethoxycarbonylpiperidine in the presence of base. The product, compound **3**, which was obtained through chromatography was subsequently reacted with *ortho*- and (or) *para*-substituted benzylamines with heating to give compounds **4**. Treatment of compounds **4** with a mixture of 10% NaOH aqueous solution and ethanol under reflux for 12 h provided the deprotected intermediates **5**. Compounds **5** were condensed with a series of commercially available carboxylic acids to afford the desired target compounds **6a**–**e**. The compounds **7a** and **7b** were synthesized in one step from compound **1** (Scheme 1). The preparation of compounds **12a**–**d** is summarized in Scheme 2. 2,4-Dichloro-6-methylpyrimidine (**8**) was reacted with 2,4-dichlorobenzylamine to give intermediate **9**, which was coupled with N-ethoxycarbonylpiperidine and the ethoxycarbonyl group was then deprotected to obtain compound **11**, which was reacted with different carboxylic acids to afford compounds **12a**–**d**.

### 2.2. Chemotaxis Activity Assay and Discussion

All the newly synthesized compounds were evaluated by a CCR4-MDC chemotaxis inhibition assay. Compound **1** was employed as the positive control. The activity results are summarized in Table 1. All of the newly synthesized compounds except **6a** and **6b** exhibited some chemotaxis inhibition activity against CCR4.

*Reagents and Conditions:* (a) N-ethoxycarbonylpiperidine, DIEA, DCM, r.t., 2 h, 24%; (b) Substituted benzylamine, DIEA, NMP, 90 °C, 12 h; (c) 10% NaOH aq, EtOH, reflux, 24 h; (d) R_3_COOH, EDCI, HOBt, TEA, DCM, r.t.; (e) Deprotection: 4 M HCl/dioxane, DCM, r.t., 2 h; (f) Piperidine or morpholine, reflux, overnight.

*Reagents and Conditions:* (a) 2,4-dichlorobenzylamine, DIEA, DCM, reflux, 24 h, 64%; (b) DIEA, NMP, N-ethoxycarbonylpiperidine, 90 °C, 12 h; (c) 10% NaOH aq, EtOH, reflux, 24 h; (d) R_3_COOH, EDCI, HOBt, TEA, DCM, r.t., overnight; (e) Deprotection: 4 M HCl/dioxane, DCM, r.t., 2 h.

**Table 1:** Chemotaxis inhibition activity of trisubstituted pyrimidine derivatives.

Compound	IC50 (μM)
**1**	0.078 ± 0.013
**6a**	NA ^a^
**6b**	NA ^a^
**6c**	0.064 ± 0.015
**6d**	0.458 ± 0.051
**6e**	0.094 ± 0.017
**7a**	0.133 ± 0.021
**7b**	0.395 ± 0.066
**12a**	0.077 ± 0.013
**12b**	0.069 ± 0.015
**12c**	0.178 ± 0.028
**12d**	0.235 ± 0.045

^a^ NA indicates no significant inhibition.

Of these compounds, **6c**, **12a** and **12b** showed higher or similar activity compared with compound **1** (IC_50_ 0.078 μM) with IC_50_ values of 0.064, 0.077 and 0.069 μM, respectively. The SAR of the right-side carbonyl group of compound **1** was explored first through designing and synthesizing analogs **6a**–**c** and comparing them to compound **1**. Both replacement of the piperidinyl group with a pyridinyl group (compound **6a**) and removal of the nitrogen atom of piperidine (compound **6b**) did not result in significant inhibition activity. The simplification of the piperidine group (compound **6c**) resulted in similar activity compared with compound **1**. The results suggest that the saturated carbonyl group containing a nitrogen atom is crucial for maintaining the activity at this position.

We next turned our attention to the left-side substituted amide portion. Compounds **6d** and **6e** were designed so that the 2,4-dichlorobenzyl group was replaced by a 4-chlorobenzyl group and 2,4-difluoro benzyl group, respectively. Compound **6d** had significantly reduced activity and **6e** had slightly reduced activity compared with compound **1**. These results illustrate that halogen group substitutions at the 2- and 4-positions of the benzyl group are crucial for maintaining the activity.

The SAR around the 6-substituted group of the central pyrimidine ring of compound **1** was then explored. Replacement of the chlorine substituent group of the pyrimidine with a piperidinyl or morpholinyl ring afforded the corresponding compounds **7a** or **7b**. Compared with compound **1**, compounds **7a** and **7b** exhibited relatively poorer activity. This implied that small and less polar substituents are beneficial for maintaining the activity. In order to confirm further this, compound **12a** was synthesized where a methyl group was substituted at the 6-position of the pyrimidine. Its activity was similar to compound **1**.

Based on these preliminary SARs, we designed and synthesized compounds **12b**–**d**. Compound **12b** showed similar activity as compound **1**. These results further prove that the SAR conclusions above are correct. Compounds **12c** and **12d** exhibited relatively weak activity. The results illustrate that a nitrogen atom at the α-position of the right-side carbonyl group is better than at the γ-position, and replacement of the nitrogen atom of the right-side carbonyl group with oxygen significantly reduces the activity.

## 3. Experimental

### 3.1. General

Melting points were determined using a YRT-3 melting point detector (P.I.F. Tianjin University, Tianjin, China) and have been reported as uncorrected values. The ^1^H-NMR (400 MHz) and ^13^C-NMR (100 MHz) spectra were recorded using a Bruker ARX 400 spectrometer (Karlsruhe, Germany). The mass spectra were determined using an Agilent 5875(ESI) spectrometer (Palo Alto, CA, USA). The high resolution mass spectra were determined using an Agilent 6230A instrument. All solvents and reagents were purchased commercially and used without further purification.

### 3.2. Chemical Synthesis

#### 3.2.1. Procedure for the Synthesis of Compounds **6a**–**e**

*4,6-Dichloro-2-(4-ethoxycarbonylpiperazino)pyrimidine* (**3**). Compound **3** was synthesized according to a well-established literature procedure [15]. 

*6-Chloro-2-(4-ethoxycarbonylpiperazino)-N-(2,4-dichlorobenzyl)pyrimidin-4-amine* (**4a**). 2,4-Dichlorobenzylamine (1.76 g, 10.0 mmol) and DIEA (2.60 g, 20.0 mmol) were added to a solution of compound **3** (3.05 g, 10.0 mmol) in NMP (30 mL), and the resulting mixture was stirred at 90 °C for 12 h. The reaction mixture was cooled to room temperature. Water was added, and the resulting solution was extracted with ethyl acetate. The organic layer was washed with brine, dried over sodium sulfate, filtered and concentrated *in vacuo*. The residue was purified by silica gel column chromatography (hexane/ethyl acetate *v*/*v* = 5:2) to obtain compound **4a** (4.12 g, 92.6%) as a white solid. ^1^H-NMR (CDCl_3_) δ ppm: 7.41 (1H, d, *J* = 1.96 Hz), 7.27 (1H, d, *J* = 8.6 Hz), 7.23 (1H, d, *J* = 8.4 Hz), 5.73 (1H, s), 5.10 (1H, brs), 4.57 (2H, s), 4.20–4.14 (2H, q), 3.74 (4H, m), 3.49 (4H, m), 1.30–1.26 (3H, t); ESI-MS: *m/z* = 444.3 [M+H]^+^.

*6-Chloro-2-(4-ethoxycarbonylpiperazino)-N-(*4-chlorobenzyl*)pyrimidin-4-amine* (**4b**). Compound **4b** was synthesized from compound **3** and 4-chlorobenzylamine according to the procedure described for the synthesis of compound **4a**. It was obtained as a white solid. ^1^H-NMR (CDCl_3_) δ ppm: 7.32 (2H, d, *J* = 8.4 Hz), 7.24 (2H, d, *J* = 8.4 Hz), 5.73 (1H, s), 5.14 (1H, brs), 4.50 (2H, s), 4.19–4.14 (2H, q), 3.75 (4H, m), 3.62 (4H, m), 1.30–1.26 (3H, t); ESI-MS: *m/z* = 410.4 [M+H]^+^.

*6-Chloro-2-(4-ethoxycarbonylpiperazino)-N-(2,4-difluorobenzyl)pyrimidin-4-amine* (**4c**). Compound **4c** was synthesized from Compound **3** and 2,4-difluorobenzylamine according to the procedure described for the synthesis of compound **4a**. It was obtained as a white solid. ^1^H-NMR (CDCl_3_) δ ppm: 7.30 (1H, m), 6.87–6.80 (2H, m), 5.74 (1H, s), 5.04 (1H, brs), 4.54 (2H, s), 4.20–4.14 (2H, q), 3.76 (4H, m), 3.49 (4H, m), 1.30–1.25 (3H, t); ESI-MS: *m/z* = 412.4 [M+H]^+^.

*6-Chloro-2-piperazino-N-(*2,4-dichlorobenzyl*)pyrimidin-4-amine* (**5a**). A mixture of compound **4a** (3.78 g, 8.5 mmol), 10% NaOH aqueous solution (40 mL) and ethanol (45 mL) was stirred at reflux for 24 h. The reaction mixture was concentrated *in vacuo*. Water was added to the residue, and the resulting mixture was extracted with dichloromethane. The organic layer was washed with brine, dried over sodium sulfate, filtered and concentrated *in vacuo*. The residue was washed with hexane to give compound **5a** (3.02 g, 95.5%) as an off-white solid. This solid was used in a subsequent reaction without further purification. ^1^H-NMR (CDCl_3_) δ ppm: 7.40 (1H, d, *J* = 1.96 Hz), 7.27 (1H, d, *J* = 8.6 Hz), 7.22 (1H, d, *J* = 8.4 Hz), 5.68 (1H, s), 5.17 (1H, brs), 4.57 (2H, s), 3.72 (4H, m), 2.87 (4H, m), 1.88 (1H, brs); ESI-MS: *m/z* = 372.0 [M+H]^+^.

*6-Chloro-2-piperazino-N-(*4-chlorobenzyl*)pyrimidin-4-amine* (**5b**). Compound **5b** was obtained as an off-white solid (2.75 g, 95.4%) from compound **4b** according to the procedure given for the synthesis of compound **5a**. ^1^H-NMR (CDCl_3_) δ ppm: 7.30 (2H, d, *J* = 8.4 Hz), 7.22(2H, d, *J* = 8.4 Hz), 5.67 (1H, s), 5.14 (1H, brs), 4.50 (2H, s), 3.72 (4H, m), 2.86 (4H, m), 1.88 (3H, brs); ESI-MS: *m/z* = 338.1 [M+H]^+^.

*6-Chloro-2-piperazino-N-(*2,4-difluorobenzyl*)pyrimidin-4-amine* (**5c**). Compound **5c** was obtained as an off-white solid (2.73 g, 95.0%) from compound **4c** according to the procedure described for the synthesis of compound **5a**. ^1^H-NMR (CDCl_3_) δ ppm: 7.29 (1H, m), 6.84 (2H, m), 5.74 (1H, s), 5.04 (1H, brs), 4.54 (2H, s), 3.73 (4H, m), 2.87 (4H, m), 1.89 (1H, brs); ESI-MS: *m/z* = 440.1 [M+H]^+^.

*6-Chloro-2-[4-(pyridin-2-yl)carbonylpiperazino]-N-(*2,4-dichlorobenzyl*)pyrimidin-4-amine* (**6a**). A mixture of compound **5a** (0.37 g, 1.0 mmol), EDCI (0.23 g, 1.2 mmol), HOBt (0.16 g, 1.2 mmol), pyridine-2-carboxylic acid (0.13 g, 1.0 mmol), TEA (1 mL) and dichloromethane (20 mL) was stirred at room temperature overnight. The reaction mixture was concentrated *in vacuo*. Water was added to the residue, and the resulting mixture was extracted with ethyl acetate. The organic layer was washed with brine, dried over sodium sulfate, filtered and concentrated *in vacuo*. The residue was purified by silica gel column chromatography (hexane/ethyl acetate/methanol) to obtain compound **6a** (0.38 g, 79.5%) as a white solid. M.p. 175.1–176.9 °C; ^1^H-NMR (CDCl_3_) δ ppm: 8.59 (1H, d, *J* = 4.7 Hz), 7.81 (1H, m, *J*_1_= 7.5 Hz, *J*_2_ = 1.6 Hz), 7.68 (1H, m), 7.38 (2H, m), 7.25(1H, d, *J* = 8.4 Hz), 7.21 (1H, dd, *J*_1_ = 8.4 Hz, *J*_2_ = 1.96 Hz), 5.73 (1H, s), 5.17 (1H, brs), 4.56 (2H, s), 3.87–3.62 (8H, m); ^13^C-NMR (CDCl_3_): δ 167.6, 163.3, 160.7, 159.7, 153.7, 148.3, 137.1, 134.2, 133.7, 129.9, 129.3, 127.1, 124.6, 123.9, 92.6, 46.9, 44.1, 43.4, 42.2; ESI-MS: *m/z* = 477.0 [M+H]^+^; HRMS (TOF): calcd for C_21_H_19_Cl_3_N_6_O [M+Na]^+^: 499.0579, Found: 499.0580.

*6-Chloro-2-(4-cyclohexylcarbonylpiperazino)-N-(*2,4-dichlorobenzyl*)pyrimidin-4-amine* (**6b**). Compound **6b** was obtained as a white solid (0.41 g, 85.4%) from compound **5a** and cyclohexanecarboxylic acid according to the procedure described for the synthesis of compound **6a**. M.p. 175.3–177.1 °C; ^1^H-NMR (CDCl_3_) δ ppm: 7.41 (1H, d, *J* = 1.96 Hz), 7.29 (1H, d, *J* = 8.4 Hz), 7.23 (1H, dd, *J*_1_= 8.4 Hz, *J*_2_= 1.96 Hz), 5.74 (1H, s), 5.15(1H,brs), 4.58 (2H, s), 3.76–3.51 (8H, m), 2.48 (1H, m), 1.82–1.25 (10H, m); ^13^C-NMR (CDCl_3_): δ 174.8, 163.4, 160.7, 133.8, 129.9, 129.4, 127.2, 92.8, 45.0, 44.1, 43.6, 42.4, 41.3, 40.4, 29.3, 25.7; ESI-MS: *m/z* = 482.3 [M+H]^+^; HRMS (TOF): calcd for C_22_H_26_Cl_3_N_5_O [M+Na]^+^: 504.1101, Found: 504.1009.

*6-Chloro-2-[4-(N,N-dimethylaminomethyl)carbonylpiperazino]-N-(2,4-dichlorobenzyl)pyrimidin-4-amine* (**6c**). Compound **6c** was obtained as a white solid (0.30 g, 65.4%) from compound **5a** and 2-(dimethylamino)acetic acid according to the procedure given for the synthesis of compound **6a**. M.p. 166.5–168.5 °C; ^1^H-NMR (CDCl_3_) δ ppm: 7.41 (1H, d, *J* = 1.96 Hz), 7.27 (1H, d, *J* = 8.4 Hz), 7.23 (1H, dd, *J*_1_= 8.4 Hz, *J*_2_= 1.96 Hz), 5.74 (1H, s), 5.15(1H,brs), 4.58 (2H, s), 3.76 (4H, m), 3.63 (4H, m), 3.21 (2H, s), 2.35 (6H, s); ^13^C-NMR (CDCl_3_): δ 168.6, 163.3, 160.7, 133.7, 133.6, 129.9, 129.2, 127.1, 92.4, 62.5, 45.4, 45.2, 44.0, 43.5, 42.3, 41.5; ESI-MS: *m/z* = 457.0 [M+H]^+^; HRMS (TOF): calcd for C_19_H_23_Cl_3_N_6_O [M+H]^+^: 457.1072, Found: 457.1071.

*(R)-6-Chloro-2-[4-(piperidin-2-yl)carbonylpiperazino]-N-(*4-chlorobenzyl*)pyrimidin-4-amine* (**6d**). A mixture of compound **5b** (0.51 g, 1.5 mmol), EDCI (0.35 g, 1.8 mmol), HOBt (0.24 g, 1.8 mmol), (R)-*N*-piperidine-2-carboxylic acid (0.35 g, 1.5 mmol), TEA (1.5 mL) and dichloromethane (30 mL) was stirred at room temperature overnight. The reaction mixture was concentrated *in vacuo*. Water was added to the residue, and the resulting mixture was extracted with ethyl acetate. The organic layer was washed with brine, dried over sodium sulfate, filtered, and concentrated *in vacuo*. The residue (0.74 g) was dissolved in dichloromethane (10) and 4 M HCl in dioxane (0.5 mL) was added dropwise. The reaction mixture was stirred at room temperature for 1 h. Then the pH of reaction mixture was adjusted to 10 with a 10% NaOH aqueous solution. The resulting mixture was extracted with dichloromethane. The organic layer was washed with brine, dried over sodium sulfate, filtered and concentrated in vacuo. Ethyl acetate was added to the crude residue, stirred and filtered. The solid was washed with additional ethyl acetate and dried to obtain compound **6d** (0.47 g, 70.6%) as a white solid. M.p. 100.3–101.9 °C; ^1^H-NMR (CDCl_3_) δ ppm: 7.30 (2H, d, *J* = 8.4 Hz), 7.24 (2H, d, *J* = 8.4 Hz), 5.74 (1H, s), 5.24 (1H, m), 4.48 (2H, s), 3.78–3.53 (8H, m), 3.43 (1H, m), 3.27 (1H, d), 2.80–2.74 (1H, m), 1.91 (1H, d), 1.76 (1H, d), 1.63 (1H, d), 1.55–1.47 (3H, m); ^13^C-NMR (CDCl_3_): δ 171.2, 163.4, 160.7, 136.9, 133.0, 128.7, 128.6, 92.7, 56.1, 45.1, 45.0, 44.4, 43.8, 43.4, 41.6, 29.3, 25.5, 23.8; ESI-MS: *m/z* = 449.5 [M+H]^+^; HRMS (TOF): calcd for C_22_H_28_Cl_2_N_6_O [M+H]^+^: 449.1618, Found: 449.1618.

*(R)-6-Chloro-2-[4-(piperidin-2-yl)carbonylpiperazino]-N-((2,4-difluorobenzyl)pyrimidin-4-amine* (**6e**). Compound **6c** was obtained as a white solid (0.44 g, 65.4%) from compound **5c** according to the procedure described for the synthesis of compound **6d**. M.p. 87.2–89.0 °C; ^1^H-NMR (CDCl_3_) δ ppm: 7.28 (1H, m), 6.84 (2H, m), 5.77 (1H, s), 5.11 (1H, m), 4.54 (2H, s), 3.79–3.47 (9H, m), 3.16 (1H, d), 2.76 (1H, brs), 2.71–2.65 (1H, m), 1.92 (1H, d), 1.73 (1H, d), 1.58 (1H, d), 1.50–1.45 (3H, m); ^13^C-NMR (CDCl_3_): δ 172.1, 163.3, 162.0, 161.0, 160.7, 159.5, 130.3, 111.3, 111.1, 103.8, 92.6, 56.4, 45.9, 45.6, 44.9, 43.8, 43.4, 41.5, 38.4, 30.0, 26.6, 24.3; ESI-MS: *m/z* = 451.3 [M+H]^+^; HRMS (TOF): calcd for C_21_H_25_ClF_2_N_6_O [M+H]^+^: 451.1819, Found: 451.1816.

#### 3.2.2. Procedure for the Synthesis of Compounds **7a** and **7b**

*(R)-2-[4-(Piperidin-2-yl)carbonylpiperazino]-N-(2,4-dichlorobenzyl)-6-piperidino pyrimidin-4-amine* (**7a**). A mixture of compound **1** (0.48 g, 1.0 mmol) and piperidine (5 mL) was stirred at reflux overnight. Water was added, and the resulting solution was extracted with ethyl acetate. The organic layer was washed with brine, dried over sodium sulfate, filtered, and concentrated *in vacuo*. The residue was purified by silica gel column chromatography (hexane/ethyl acetate/methanol) to obtain compound **7a** (0.28 g, 52.6%) as an off-white solid. M.p. 147.1–149.1 °C; ^1^H-NMR (CDCl_3_) δ ppm: 7.38 (1H, d, *J* = 1.96 Hz), 7.32 (1H, d, *J* = 8.4 Hz), 7.19 (1H, dd, *J*_1_ = 8.4 Hz, *J*_2_ = 1.96 Hz), 4.93 (1H, s), 4.50 (2H, d), 3.77–3.55 (8H, m), 3.47 (5H, m), 3.28 (1H, d), 2.77 (1H, t), 1.92 (1H, m), 1.78 (1H, m), 1.63–1.50 (10H, m); ^13^C-NMR (CDCl_3_): δ 172.0, 163.9, 163.7, 161.0, 135.4, 133.5, 133.2, 129.9, 129.0, 127.0, 73.0, 56.4, 45.6, 45.2, 44.0, 43.6, 42.6, 41.7, 30.1, 26.6, 25.4, 24.7, 24.4; ESI-MS: *m/z* = 532.4 [M+H]^+^; HRMS (TOF): calcd for C_26_H_35_Cl_2_N_7_O [M+H]^+^: 532.2358, Found: 532.2356.

*(R)-2-[4-(Piperidin-2-yl)carbonylpiperazino]-N-(2,4-dichlorobenzyl)-6-morpholino pyrimidin-4-amine* (**7b**). Compound **7b** was obtained as an off-white solid (0.30 g, 55.4%) from compound **1** and morpholine according to the procedure described for the synthesis of compound **7a**. M.p. 167.8–169.5 °C; ^1^H-NMR (CDCl_3_) δ ppm: 7.39 (1H, d, *J* = 1.96 Hz), 7.30 (1H, d, *J* = 8.4 Hz), 7.20 (1H, dd, *J*_1_ = 8.4 Hz, *J*_2_ = 1.96 Hz), 4.92 (1H, s), 4.90 (1H, brs), 4.52 (2H, d), 3.76–3.54 (12H, m), 3.44 (5H, m), 3.28 (1H, d), 2.76 (1H, t), 1.92 (1H, m), 1.77 (1H, m), 1.63 (1H, d), 1.51–1.43 (3H, m); ^13^C-NMR (CDCl_3_): δ 172.1, 164.1, 163.9, 160.9, 135.2, 133.5, 133.2, 129.8, 129.1, 127.0, 73.3, 66.5, 56.4, 45.6, 45.1, 44.5, 44.0, 43.5, 42.5, 41.6, 30.1, 26.6, 24.4; ESI-MS: *m/z* = 534.3 [M+H]^+^; HRMS (TOF): calcd for C_25_H_33_Cl_2_N_7_O_2_ [M+H]^+^: 534.2146, Found: 534.2142.

#### 3.2.3. Procedure for the Synthesis of Compounds **12a**–**d**

*2-Chloro-N-(2,4-dichlorobenzyl)-6-methylpyrimidin-4-amine* (**9**). 2,4-Dichlorobenzylamine (7.04 g, 40.0 mmol) was dropped into a mixture of compound **8** (6.52 g, 40.0 mmol), DIEA (6.22 g, 48.0 mmol) and dichloromethane (150 mL) at room temperature. The resulting mixture was then stirred at reflux overnight. The reaction mixture was washed with distilled water and brine successively. The obtained organic layer was dried over sodium sulfate, filtered and concentrated *in vacuo*. The residue was purified by silica gel column chromatography (hexane/ethyl acetate *v/v* = 5:1) to give compound **9** (7.56 g, 62.6%) as a white solid. ^1^H-NMR (DMSO-*d_6_*) δ ppm: 8.27 (1H, brs), 7.64 (1H, d, *J* = 1.96 Hz), 7.43–7.41 (1H, dd, *J*_1_ = 8.4 Hz, *J*_2_ = 1.96 Hz), 7.34 (1H, d, *J* = 8.2 Hz), 6.40 (1H, s), 4.52 (2H, s), 2.20 (3H, s); ESI-MS: *m/z* = 302.5 [M+H]^+^.

*2-(4-Ethoxycarbonylpiperazino)-N-(2,4-dichlorobenzyl)-6-methylpyrimidin-4-amine* (**10**). Compound **10** was obtained as a white solid (5.01 g, 89.4%) from compound **9** and N-ethoxycarbonylpiperidine according to the procedure described for the synthesis of compound **4a**. ^1^H-NMR (DMSO-*d_6_*) δ ppm: 7.60 (1H, d, *J* = 1.96 Hz), 7.54 (1H, brs), 7.41–7.38 (1H, dd, *J*_1_ = 8.4 Hz, *J*_2_ = 1.96 Hz), 7.34 (1H, d, *J* = 8.4 Hz), 5.74 (1H, s), 4.48 (2H, s), 4.02–4.06 (2H, q), 3.58 (4H, m), 3.31 (4H, m), 2.06 (3H, s), 1.20–1.17 (3H, t); ESI-MS: *m/z* = 424.2 [M+H]^+^.

*2-Piperazino-N-(2,4-dichlorobenzyl)-6-methylpyrimidin-4-amine* (**11**). Compound **11** was obtained as an off-white solid (2.81 g, 95.4%) from compound **10** according to the procedure described for the synthesis of compound **5a**. ^1^H-NMR (CDCl_3_) δ ppm: 7.40 (1H, d, *J* = 1.96 Hz), 7.27 (1H, d, *J* = 8.4 Hz), 7.20 (1H, dd, *J*_1_= 8.4 Hz, *J*_2_= 1.96 Hz), 5.60 (1H, s), 5.04 (1H, brs), 4.57 (2H, s), 3.76 (4H, m), 2.89 (4H, m), 2.19 (3H, s); ESI-MS: *m/z* = 352.2 [M+H]^+^.

*(R)-2-[4-(Piperidin-2-yl)carbonylpiperazino]-N-(2,4-dichlorobenzyl)-6-methylpyrimidin-4-amine* (**12a**). Compound **12a** was obtained as a white solid (0.49 g, 71.4%) from compound **11** and (R)-*N*-piperidine-2-carboxylic acid according to the procedure described for the synthesis of compound **6d**. M.p. 82.6–84.5 °C; ^1^H-NMR (CDCl_3_) δ ppm: 7.39 (1H, d, *J* = 1.96 Hz), 7.27 (1H, d, *J* = 8.4 Hz), 7.20 (1H, dd, *J*_1_ = 8.4 Hz, *J*_2_ = 1.96 Hz), 5.59 (1H, s), 5.01 (1H, brs), 4.57 (2H, d), 3.78–3.50 (8H, m), 3.43 (1H, m), 3.23–3.19 (1H, d), 2.73–2.67 (1H, m), 2.18 (3H, s), 1.94–1.91 (1H, d), 1.76 (1H, d), 1.61 (1H, d), 1.51–1.44 (3H, m); ^13^C-NMR (CDCl_3_): δ 171.6, 166.1, 163.0, 161.4, 135.0, 133.6, 133.4, 129.9, 129.2, 127.1, 92.9, 55.9, 45.1, 43.9, 43.5, 42.2, 41.7, 29.7, 26.1, 24.1, 22.4; ESI-MS: *m/z* = 463.5 [M+H]^+^; HRMS (TOF): calcd for C_22_H_28_Cl_2_N_6_O [M+H]^+^: 463.1776, Found: 463.1774.

*2-[4-(N,N-dimethylaminomethyl)carbonylpiperazino]-N-(2,4-dichlorobenzyl)-6-methyl pyrimidin-4-amine* (**12b**). Compound **12b** was obtained as a white solid (0.28 g, 64.0%) from compound **11** and 2-(dimethylamino)acetic acid according to the procedure described for the synthesis of compound **6a**. M.p. 147.5–149.1 °C; ^1^H-NMR (DMSO-*d_6_*) δ ppm: 7.60 (1H, d, *J* = 1.96 Hz), 7.53 (1H, brs), 7.39 (1H, dd, *J*_1_ = 8.4 Hz, *J*_2_ = 1.96 Hz), 7.35 (1H, d, *J* = 8.4 Hz), 5.75 (1H, s), 4.50 (2H, s), 3.58 (4H, m), 3.47–3.40 (4H, m), 3.09 (2H, s), 2.18 (6H, s), 2.07 (3H, s); ^13^C-NMR (CDCl_3_): δ 168.5, 166.1, 162.9, 161.5, 135.0, 133.6, 133.4, 129.9, 129.2, 127.0, 92.8, 62.5, 45.4, 44.1, 43.6, 42.2, 41.6, 24.1; ESI-MS: *m/z* = 437.4 [M+H]^+^; HRMS (TOF): calcd for C_20_H_26_Cl_2_N_6_O [M+H]^+^: 437.1618, Found: 437.1617.

*2-[4-(N-methylpiperidin-4-yl)carbonylpiperazino]-N-(2,4-dichlorobenzyl)-6-methylpyrimidin-4-amine* (**12c**). Compound **12c** was obtained as a white solid (0.35 g, 73.5%) from compound **11** and 1-methylpiperidine-4-carboxylic acid according to the procedure described for the synthesis of compound **6a**. M.p. 146.4–148.0 °C; ^1^H-NMR (CDCl_3_) δ ppm: 7.40 (1H, d, *J* = 1.96 Hz), 7.28 (1H, d, *J* = 8.4 Hz), 7.19 (1H, dd, *J*_1_ = 8.4 Hz, *J*_2_ = 1.96 Hz), 5.60 (1H, s), 5.01 (1H, s), 4.57 (2H, d), 3.76 (4H, m), 3.64 (2H, m), 3.49 (2H, m), 2.94 (2H, d), 2.49 (1H, m), 2.31 (3H, s), 2.19 (3H, s), 2.05 (2H, m), 1.93 (2H, m), 1.77 (2H, m); ^13^C-NMR (CDCl_3_): δ 173.4, 166.1, 162.9, 161.4, 135.0, 133.6, 133.4, 129.8, 129.1, 127.0, 93.2, 54.9, 46.1, 45.2, 44.1, 43.6, 42.2, 41.5, 37.7, 28.4, 24.1; ESI-MS: *m/z* = 477.2 [M+H]^+^; HRMS (TOF): calcd for C_23_H_30_Cl_2_N_6_O [M+H]^+^: 477.1931, Found: 477.1934.

*(R)-2-[4-(Tetrahydrofuran-2-yl)carbonylpiperazino]-N-(2,4-dichlorobenzyl)-6-methylpyrimidin-4-amine*(**12d**). Compound **12d** was obtained as a white solid (0.39 g, 86.7%) from compound **11** and (R)-tetrahydrofuran-2-carboxylic acid according to the procedure described for the synthesis of compound **6a**. M.p. 124.3–125.8 °C; ^1^H-NMR (DMSO-*d_6_*) δ ppm: 7.60 (1H, d, *J* = 1.96 Hz), 7.53 (1H, brs), 7.39 (1H, dd, *J*_1_ = 8.4 Hz, *J*_2_ = 1.96 Hz), 7.35 (1H, d, *J* = 8.4 Hz), 5.75 (1H, s), 4.67 (1H, t), 4.50 (2H, s), 3.76 (2H, m), 3.61–3.43 (8H, m), 2.07 (3H, s), 2.00 (2H, m), 1.83 (2H, m); ^13^C-NMR (CDCl_3_): δ 170.0, 166.1, 162.9, 161.4, 135.0, 133.6, 133.4, 129.9, 129.1, 127.0, 93.1, 75.8, 69.0, 45.2, 43.9, 43.5, 42.2, 41.9, 28.4, 25.6, 24.1; ESI-MS: *m/z* = 451.3 [M+H]^+^; HRMS (TOF): calcd for C_21_H_25_Cl_2_N_5_O_2 _[M+H]^+^: 450.1458, Found: 450.1453.

### 3.3. Chemotaxis Inhibition Assay Method

The quantitative chemotaxis assays were performed using 48-well Boyden chambers (Neutroprobe, Bethesda, MD, USA) with polyvinylpyrrolidone-free polycarbonate filters with 5 μm pores [19,20]. The MDC (Peprotech, Rocky Hill, NJ, USA) with a final concentration of 10 ng/mL was added into the lower wells. The L1.2 cells that were transfected with pcDI-CCR4 were suspended in assay buffer at 3 × 10^6^ cells/mL, incubated with various final concentrations of compounds for 30 min at 37 °C, and then added to the upper wells. The chamber was incubated for 6 h at 37 °C in an atmosphere containing 5% CO_2_, and then the upper chambers were removed. The chemotaxis buffer in the lower wells was then harvested and mixed with 25 μL of the luciferase-containing reagent, Cell-Titer Glo. The number of L1.2 cells in the lower well medium was measured with a Veritas Microplate Luminometer (Turner BioSystems, Sunnyvale, CA, USA). All samples were assayed three times. The negative control samples and solvent control samples were defined as the cells migrating toward the lower wells in the absence of chemokine, and as the cells migrating toward the MDC-containing lower wells in the absence of compound in the presence of DMSO, respectively. The percentage of inhibition was determined by the following equation: inhibition = (C − A)/(C − B) × 100%, where A indicates the number of cells in the sample, B indicates the number of cells in the negative control sample and C indicates the number of cells in the solvent control sample. The results are expressed as an IC_50_ value (sample concentration that inhibits 50% chemotaxis) and calculated using Origin 8.0 software.

## 4. Conclusions

In summary, we have designed and synthesized a series of novel trisubstituted pyrimidine amide derivatives and evaluated them for their ability to inhibit CCR4-MDC chemotaxis. Compounds **6c**, **12a** and **12b** showed higher or similar activity compared with the lead compound **1**, with IC_50_ values of 0.064, 0.077 and 0.069 μM, respectively. The results from the SAR study indicated that for maintaining the activity it was critical that the right-side contain a saturated carbonyl group with a nitrogen atom and the left-side contain a benzyl group substituted with halogen at the 2- and 4-positions. The α-position nitrogen atom on the right-side carbonyl group is more beneficial for maintaining the activity than the γ-position, and replacement of the nitrogen atom with oxygen significantly reduces the activity. Small and less polar substituents on the central pyrimidine ring are beneficial for maintaining the activity. The results from the SAR study should prove helpful for identifying more potent CCR4 antagonists. Further studies on the mechanism and *in vivo* activity of these compounds are in progress. 

## Supplementary Materials

Supplementary materials can be accessed at: http://www.mdpi.com/1420-3049/19/3/3539/s1.
