# Supplementary material for: Design, Synthesis and SAR Study of Novel Trisubstituted Pyrimidine Amide Derivatives as CCR4 Antagonists
Source: Molecules. 2014 Mar 21;19(3):3539–51. doi: 10.3390/molecules19033539 (PMC6271259; doi:10.3390/molecules19033539)

Supplementary Materials

Figure S1. The ^{1}H-NMR spectrum of **6a**.

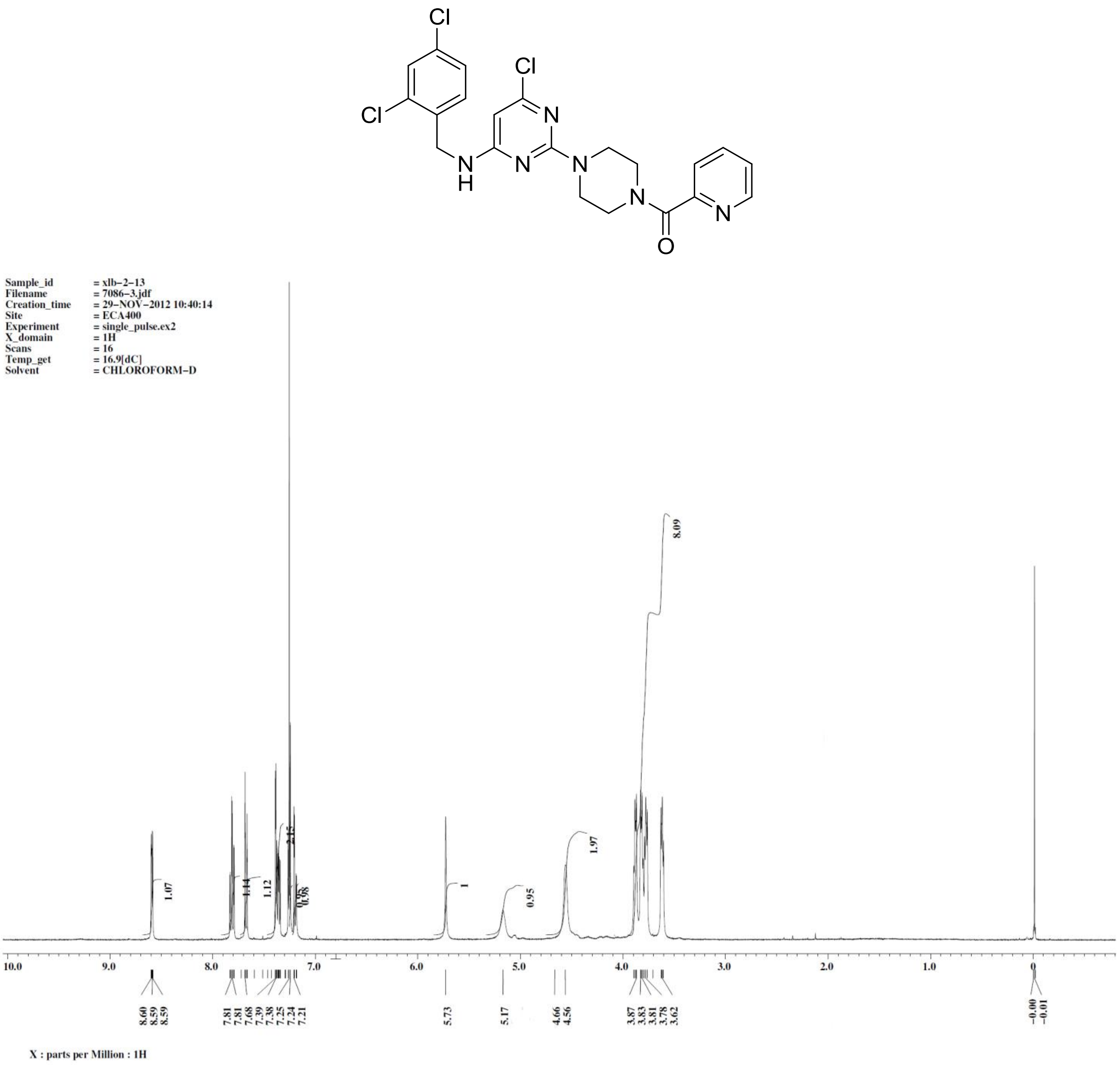

Figure S2. The ^{13}C-NMR spectrum of **6a**.

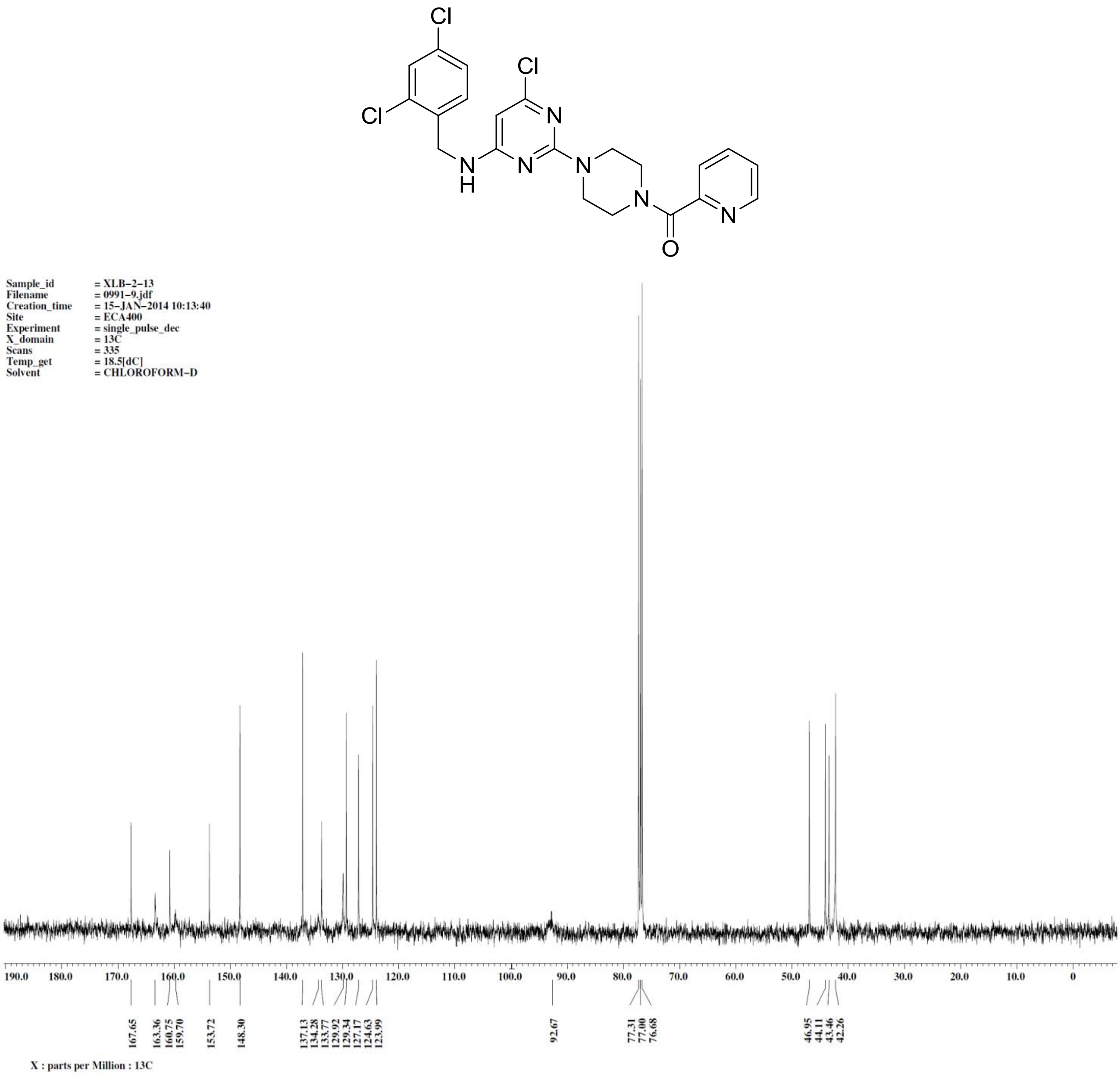

Figure S3. The ^{1}H-NMR spectrum of **6b**.

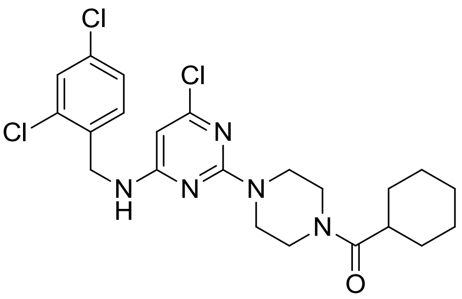

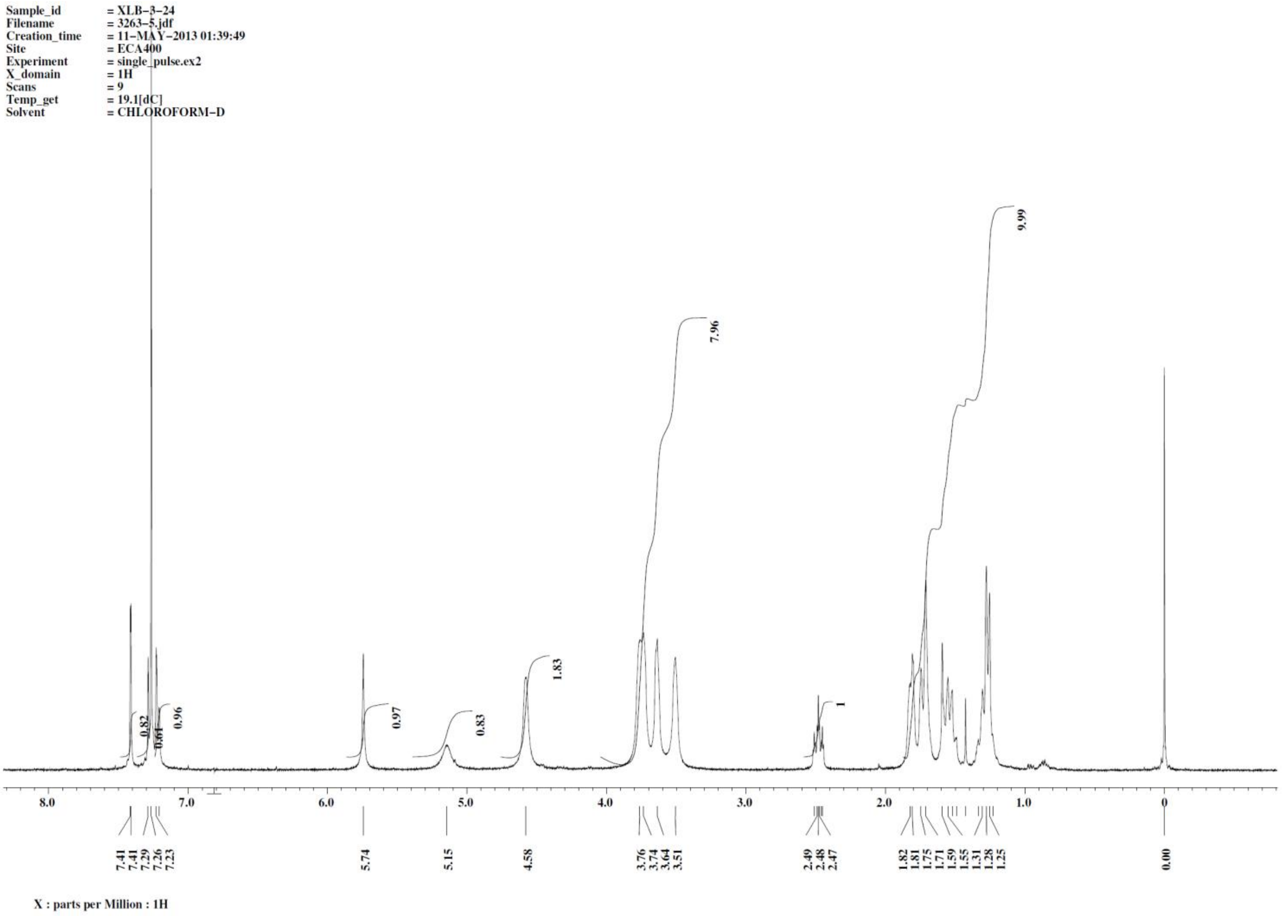

Figure S4. The ^{13}C-NMR spectrum of **6b**.

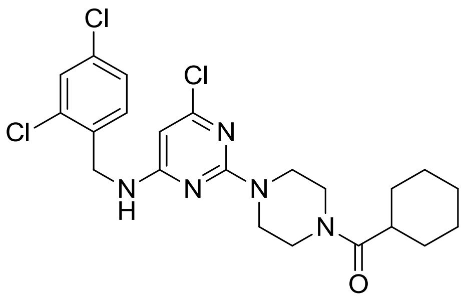

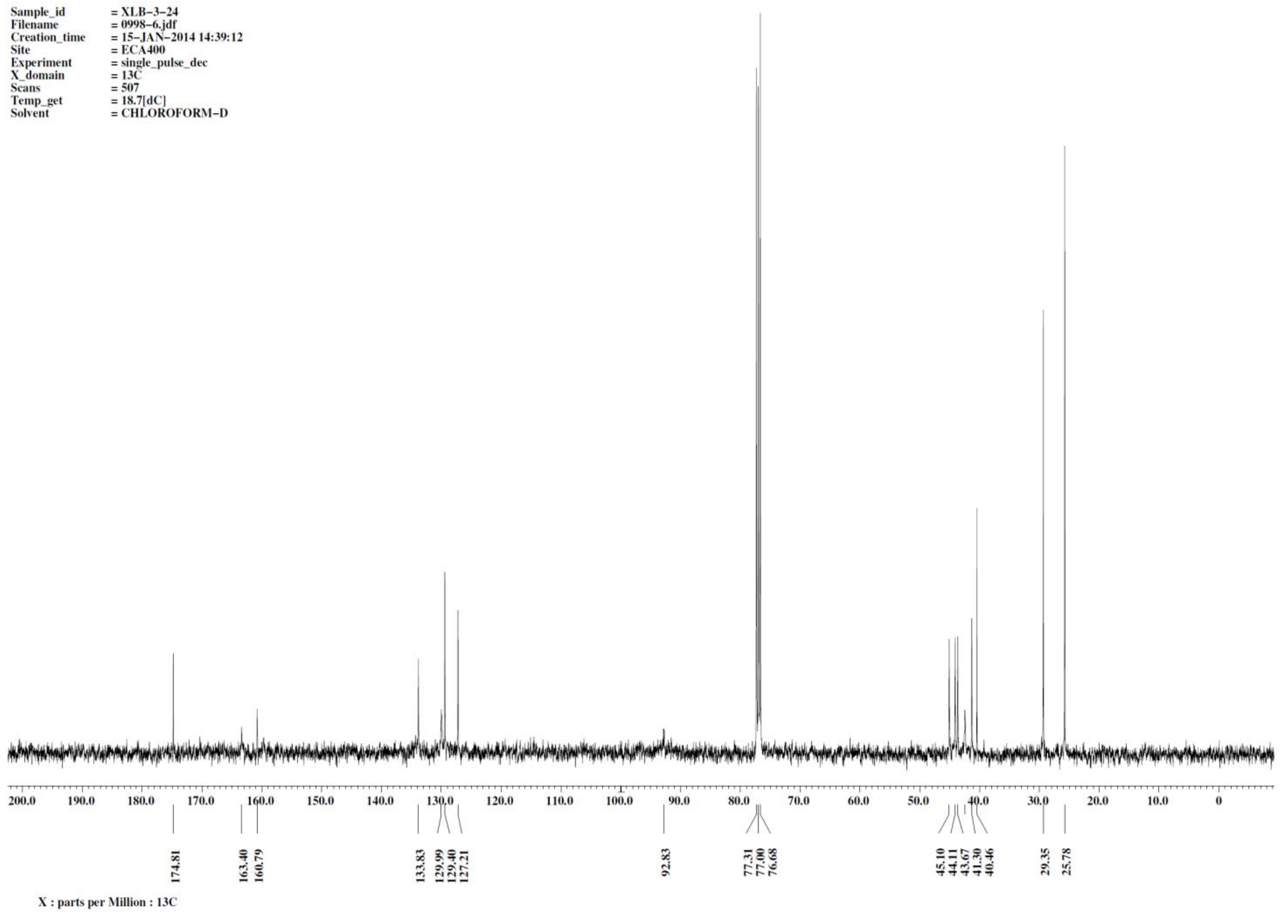

Figure S5. The ^{1}H-NMR spectrum of **6c**.

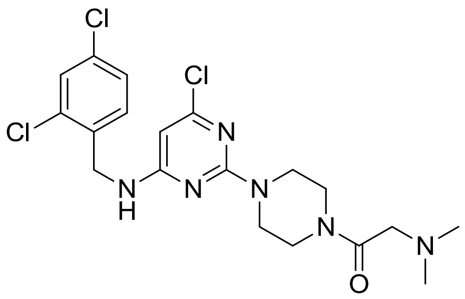

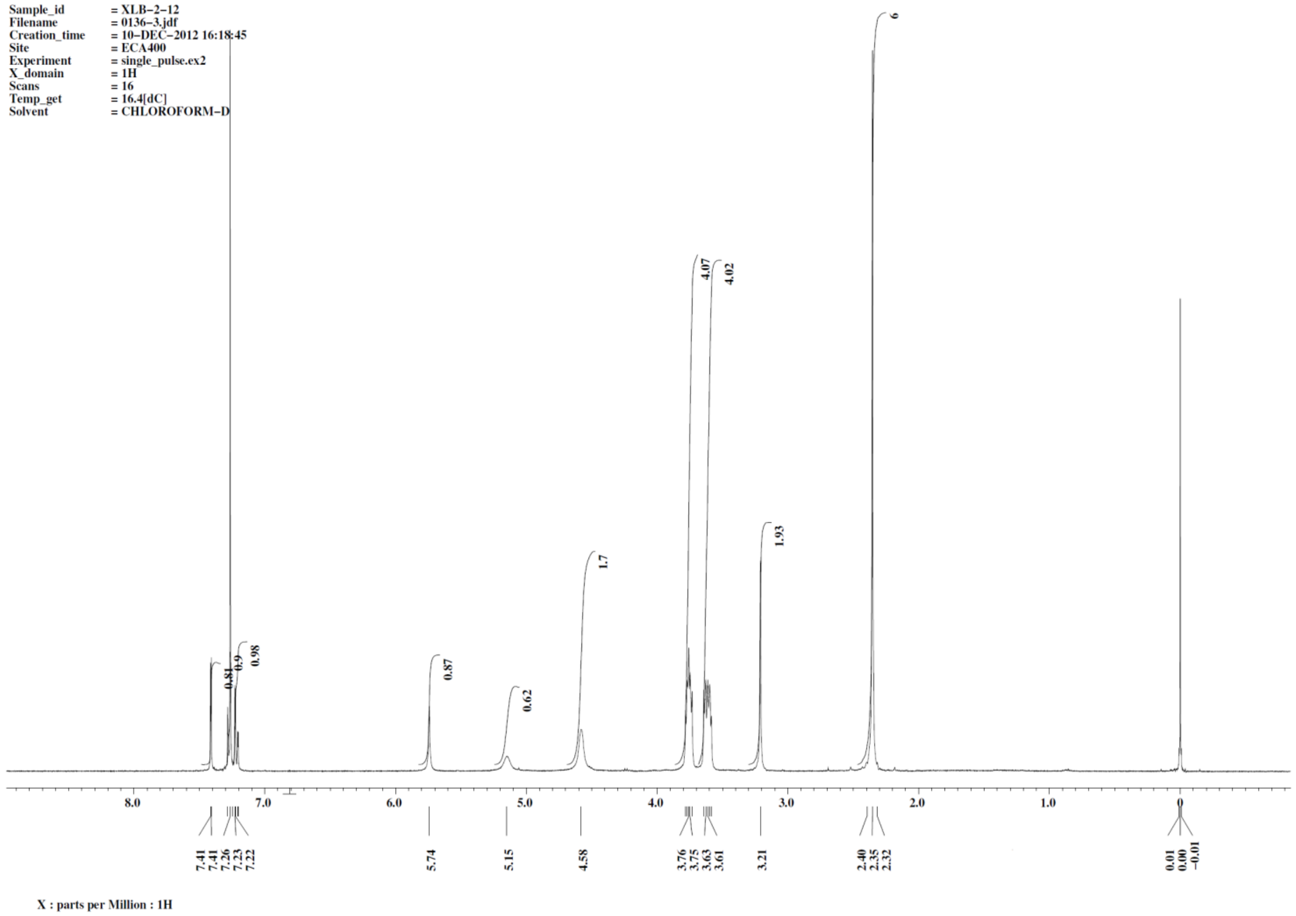

Figure S6. The ^{13}C-NMR spectrum of **6c**.

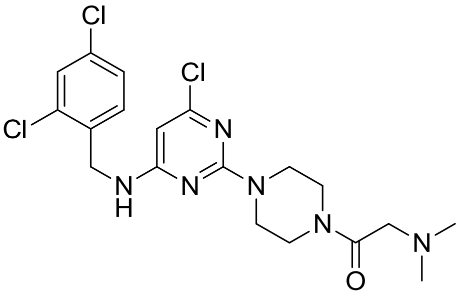

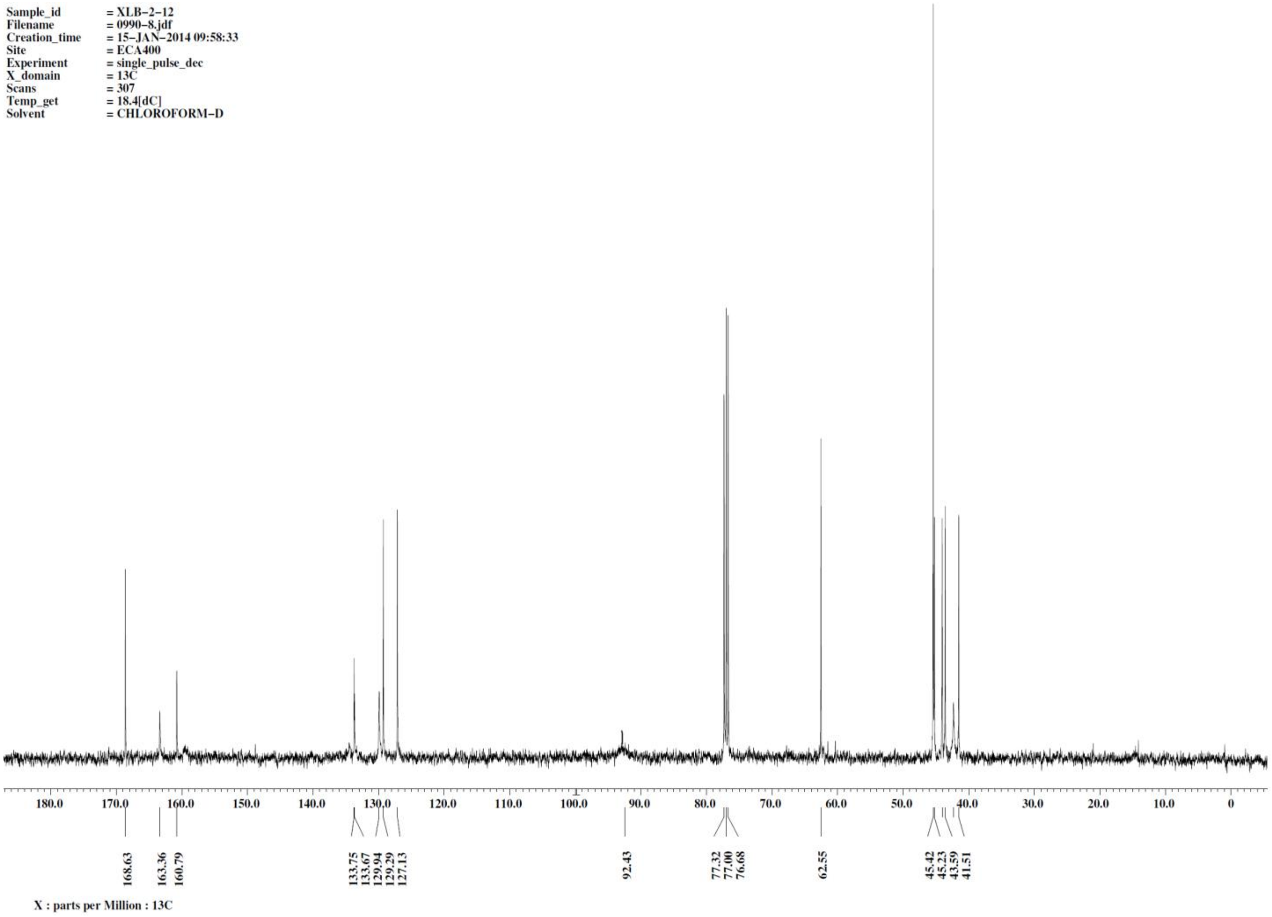

Figure S7. The ^{1}H-NMR spectrum of **6d**.

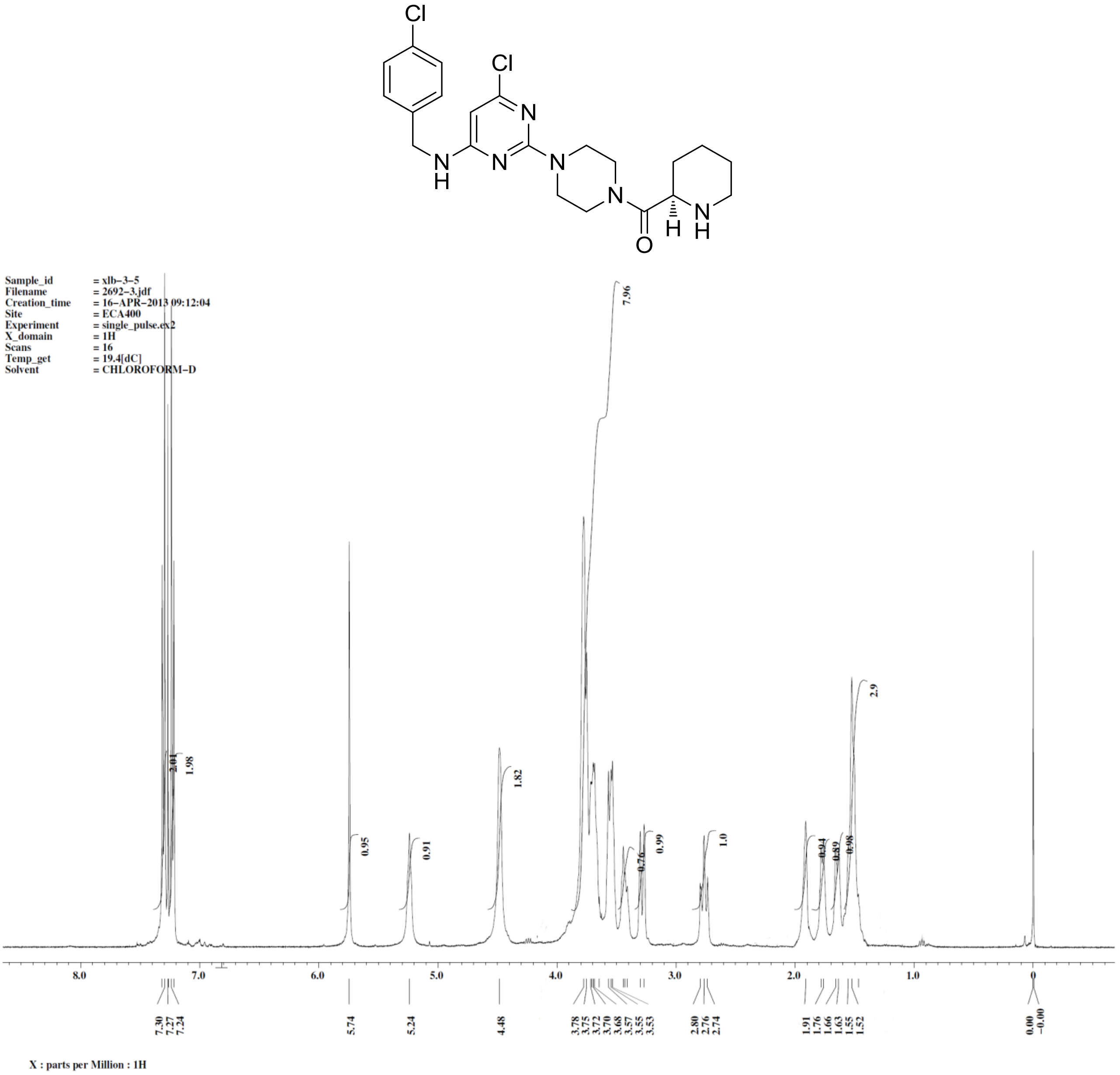

Figure S8. The ^{13}C-NMR spectrum of **6d**.

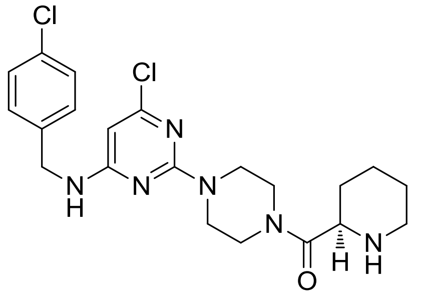

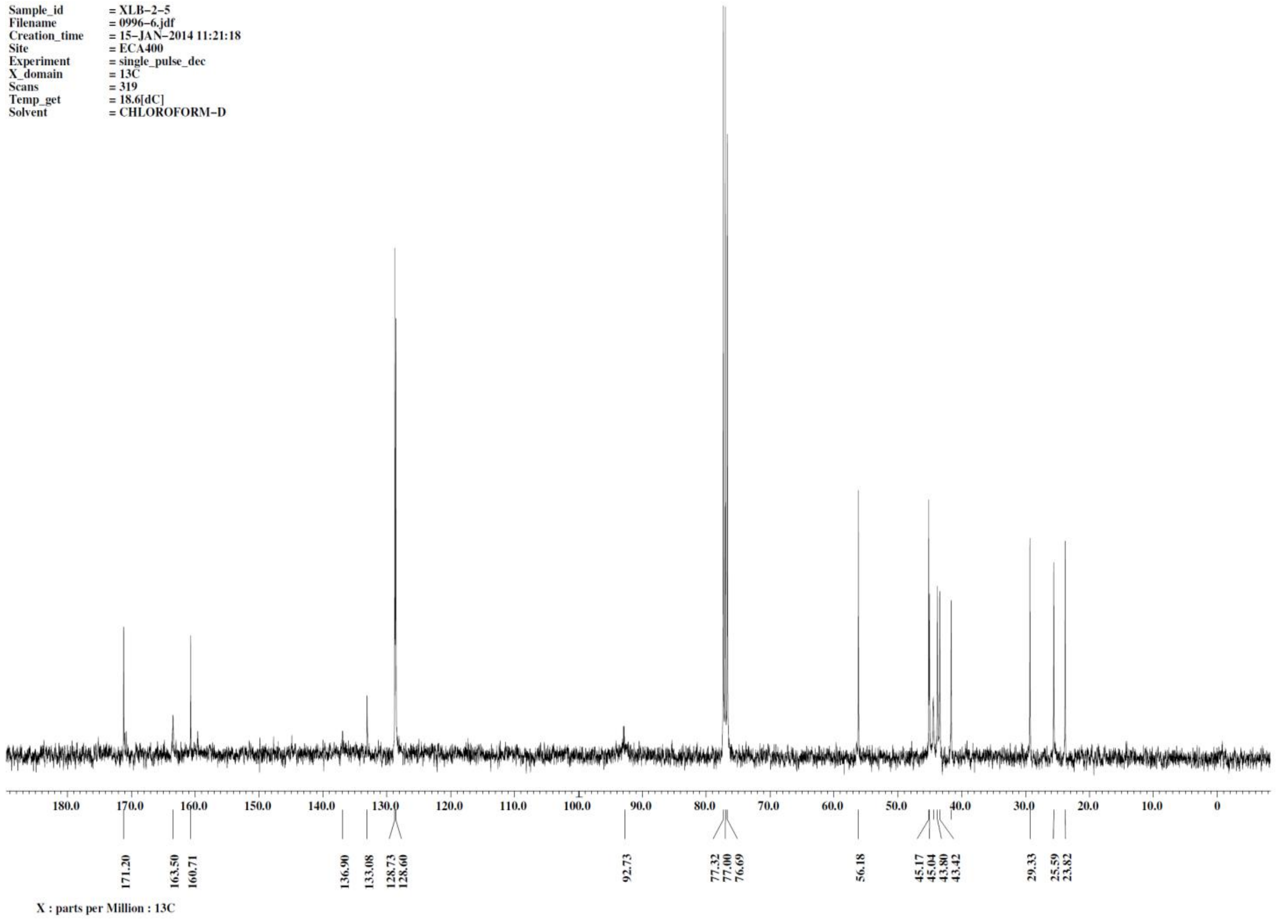

Figure S9. The ^{1}H-NMR spectrum of **6e**.

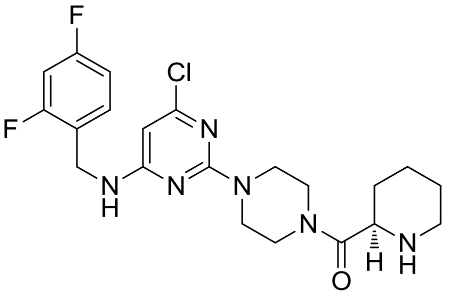

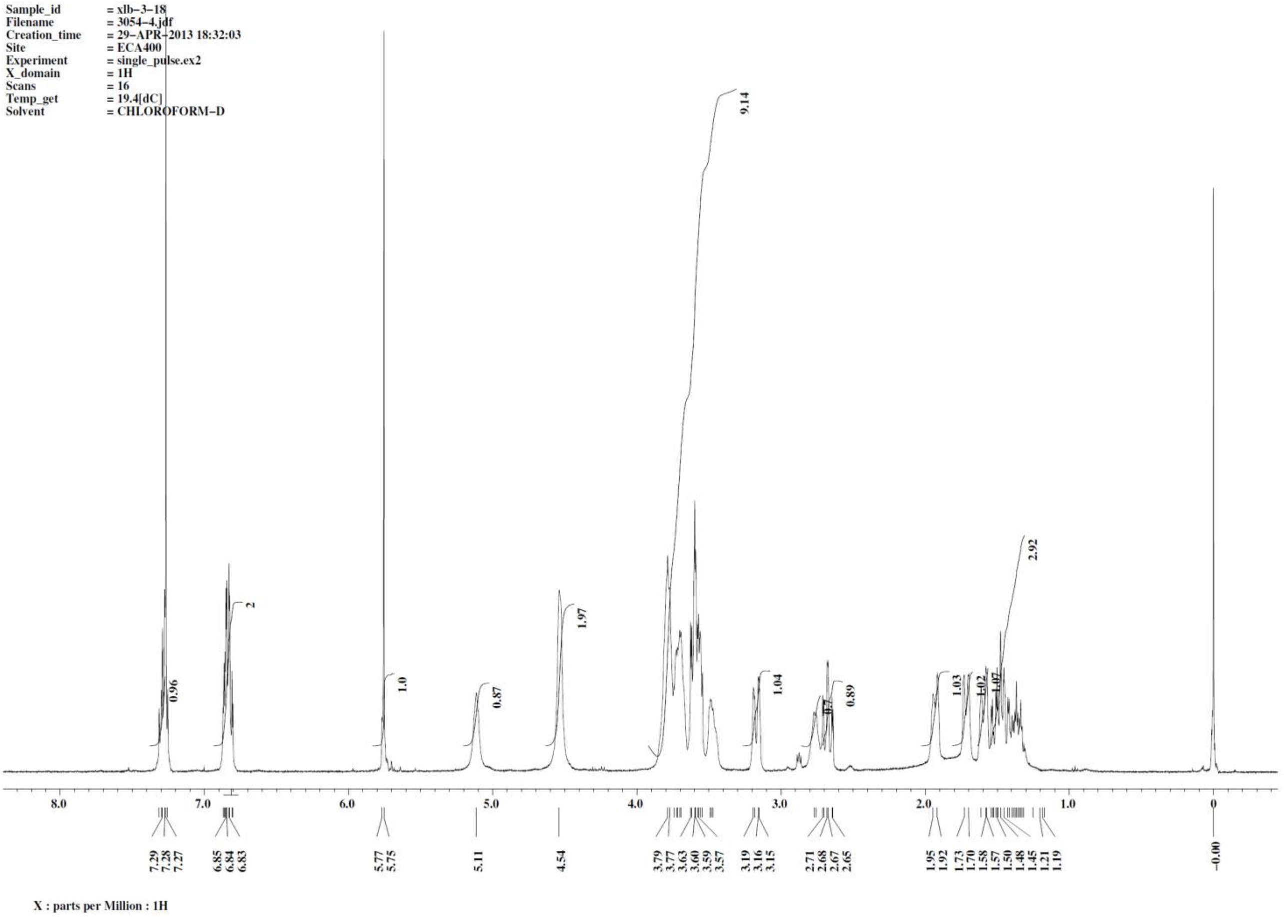

Figure S10. The ^{13}C-NMR spectrum of **6e**.

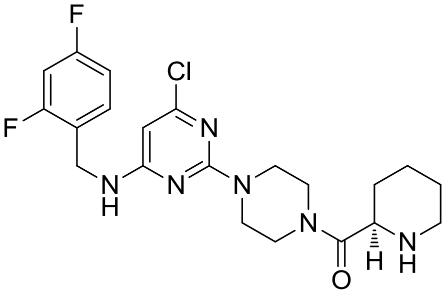

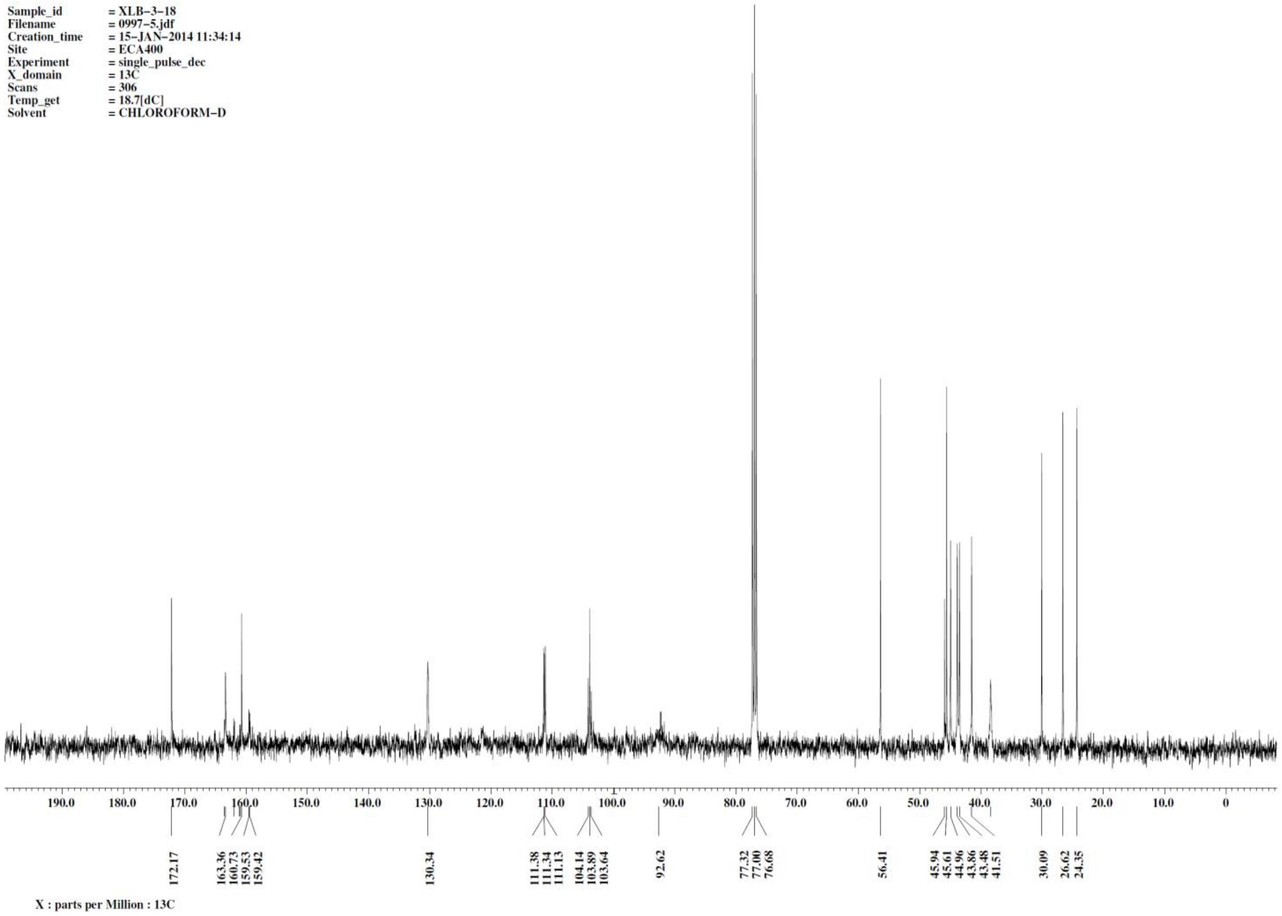

Figure S11. The ^{1}H-NMR spectrum of **7a**.

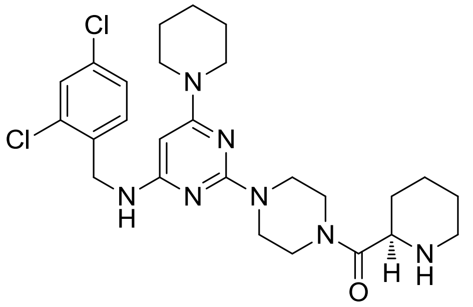

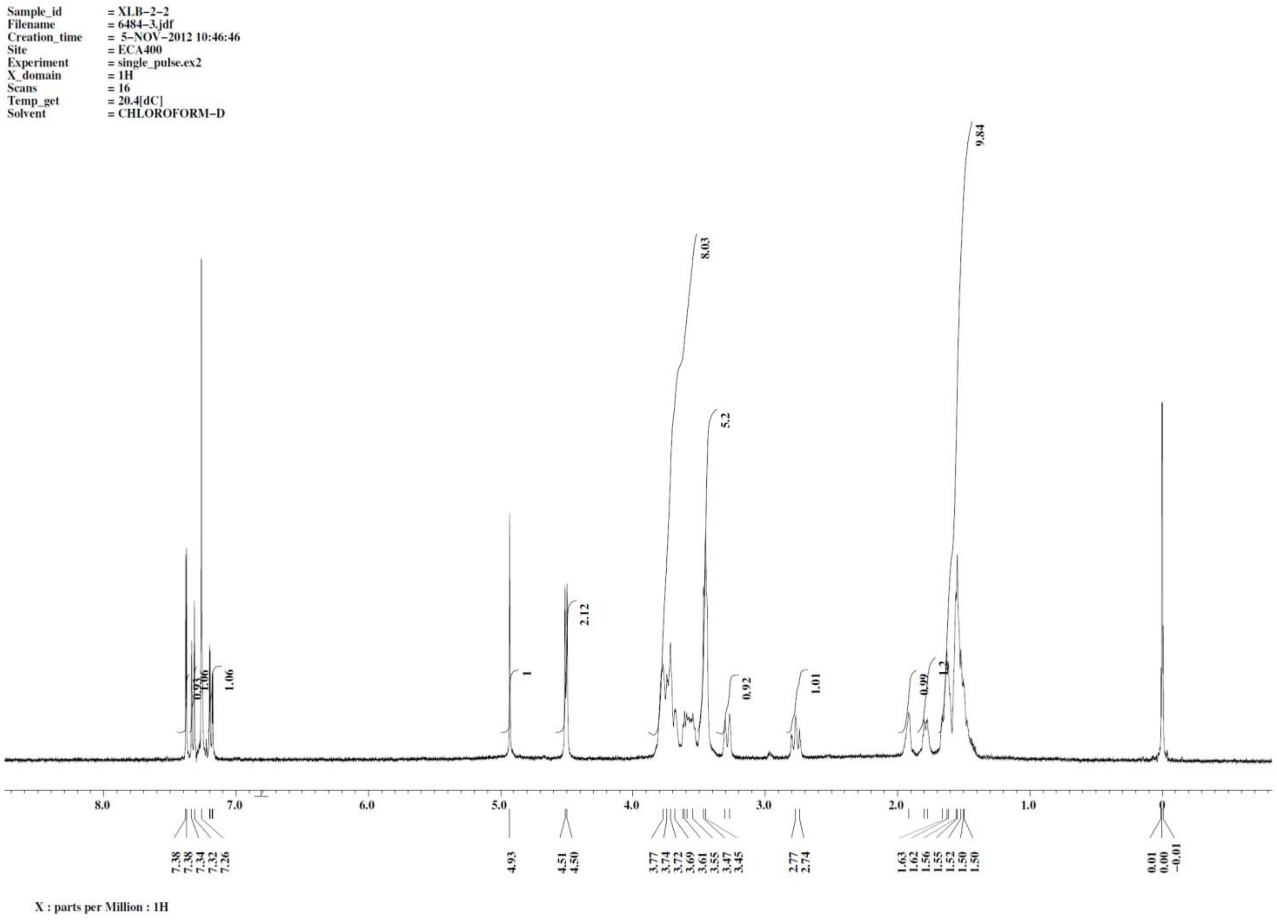

Figure S12. The ^{13}C-NMR spectrum of **7a**.

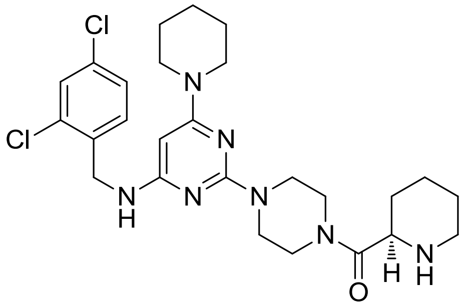

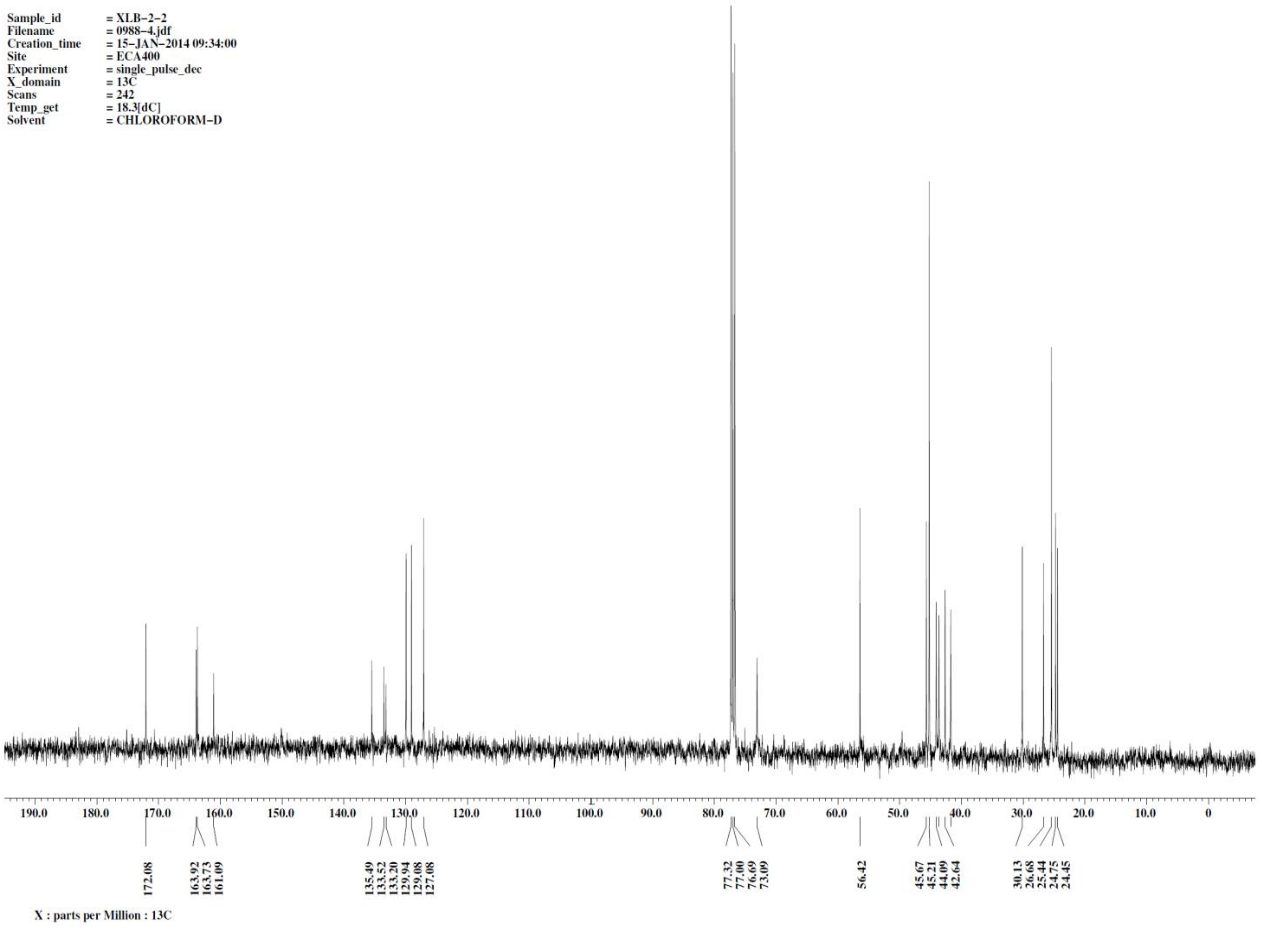

Figure S13. The ^{1}H-NMR spectrum of **7b**.

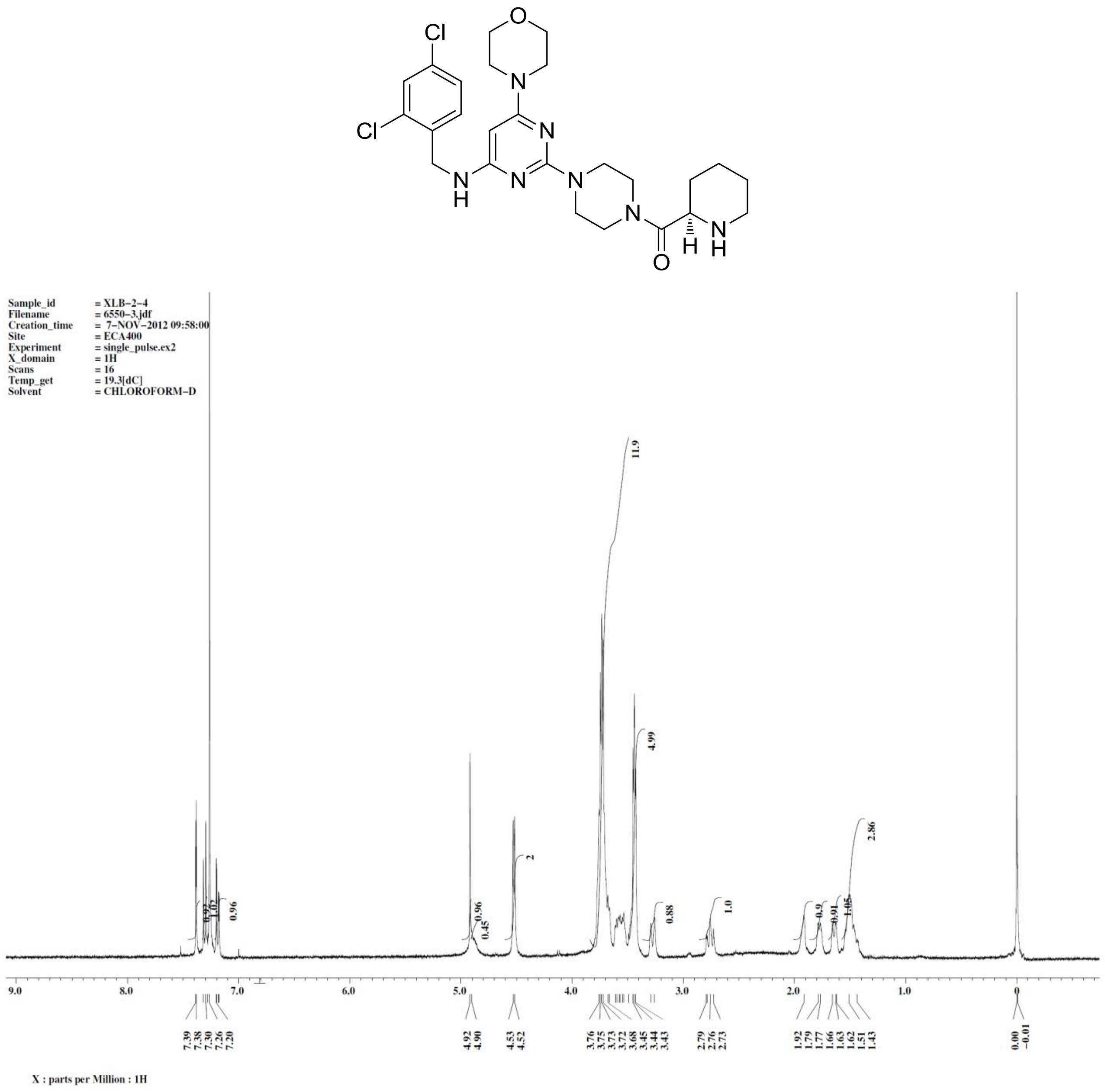

Figure S14. The ^{13}C-NMR spectrum of **7b**.

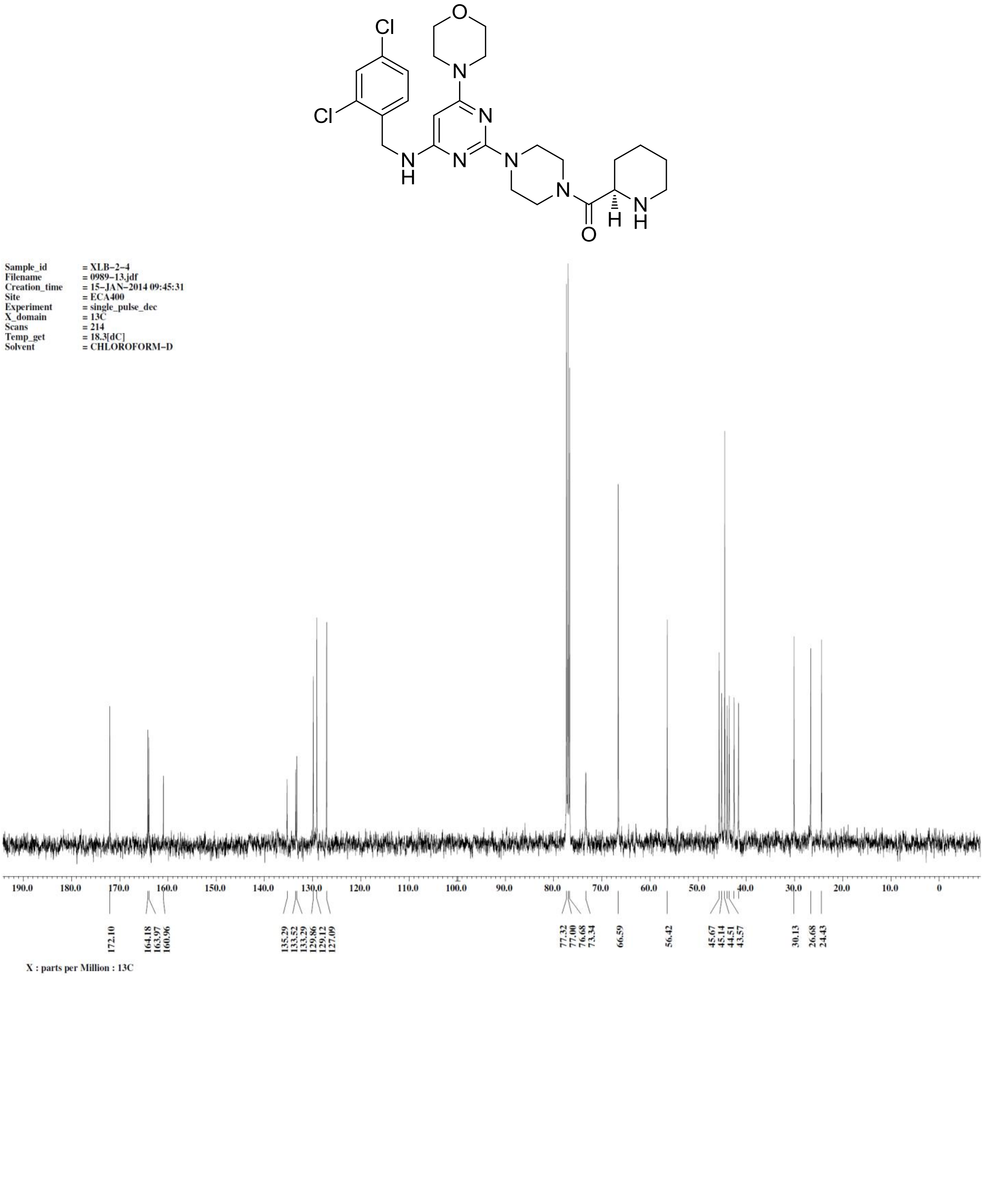

Figure S15. The ^{1}H-NMR spectrum of **12a**.

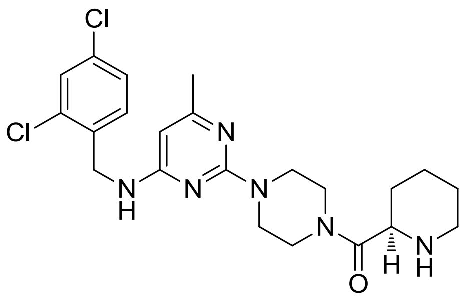

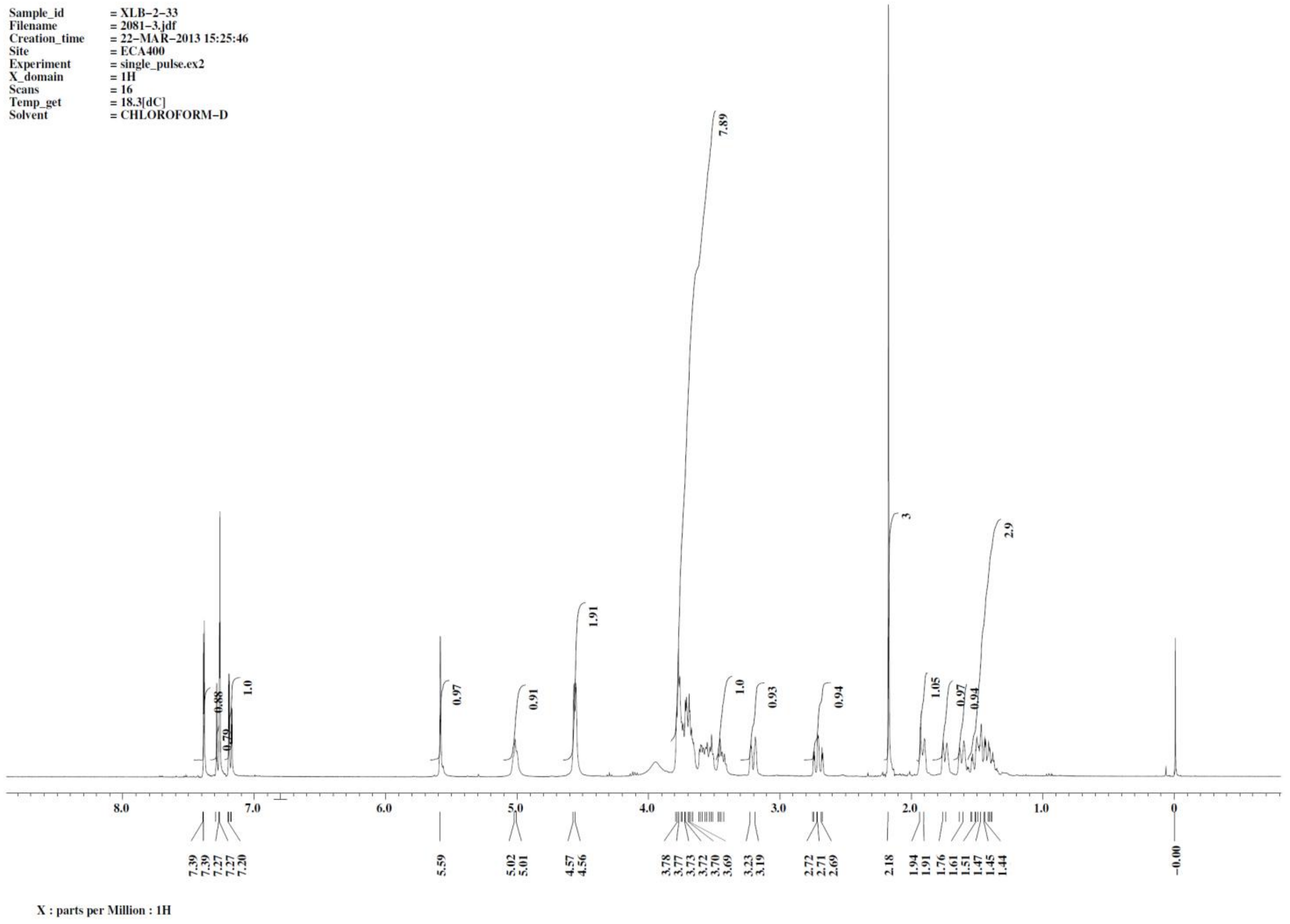

Figure S16. The ^{13}C-NMR spectrum of **12a**.

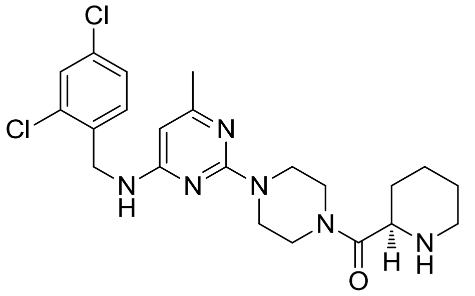

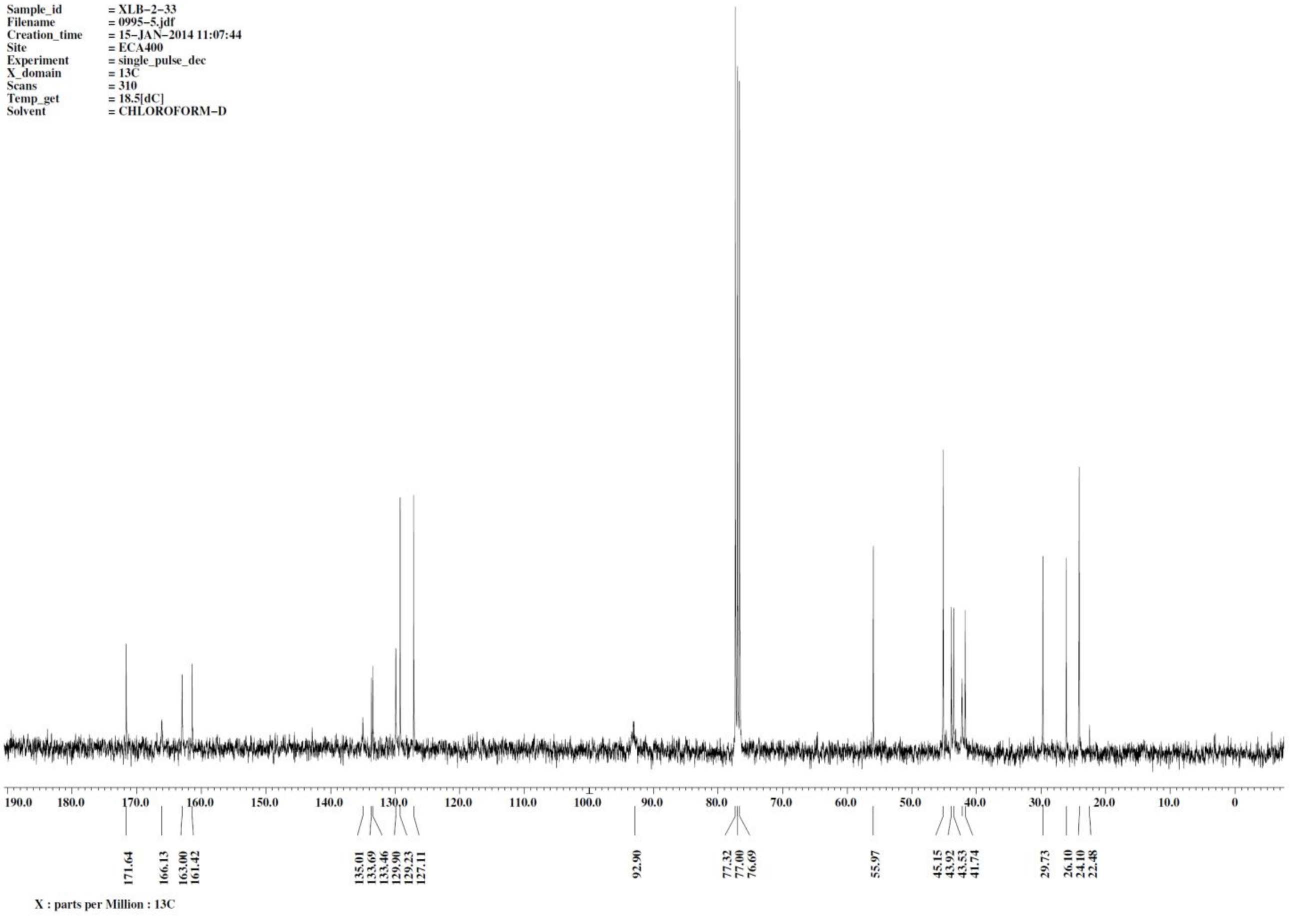

Figure S17. The ^{1}H-NMR spectrum of **12b**.

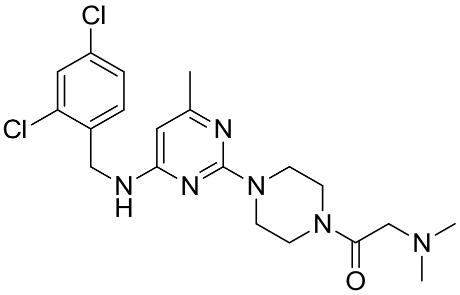

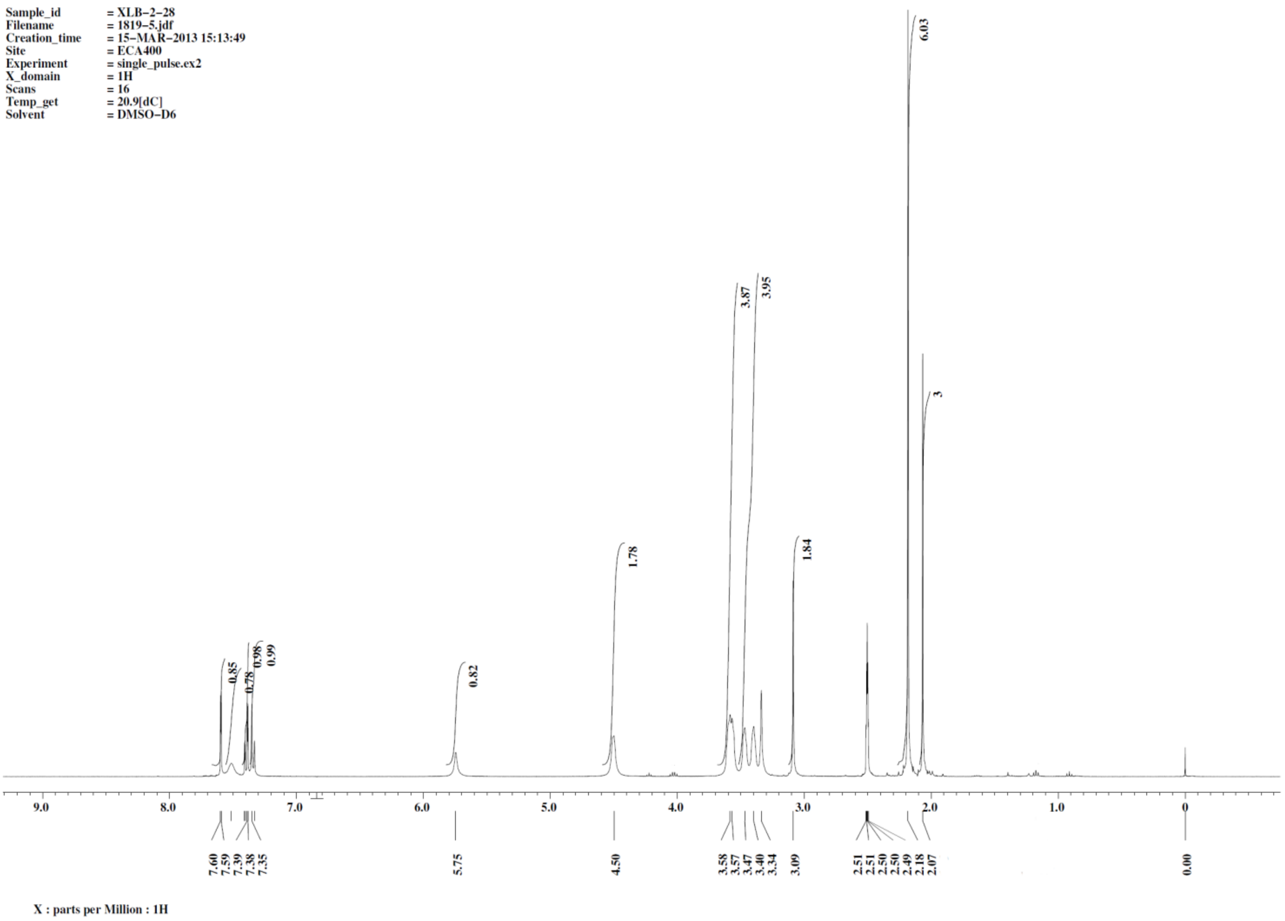

Figure S18. The ^{13}C-NMR spectrum of **12b**.

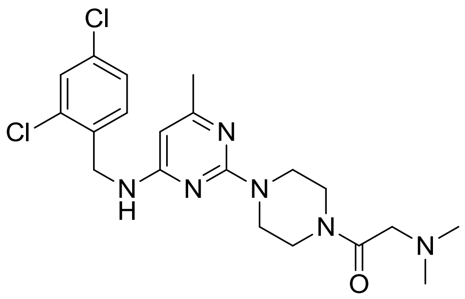

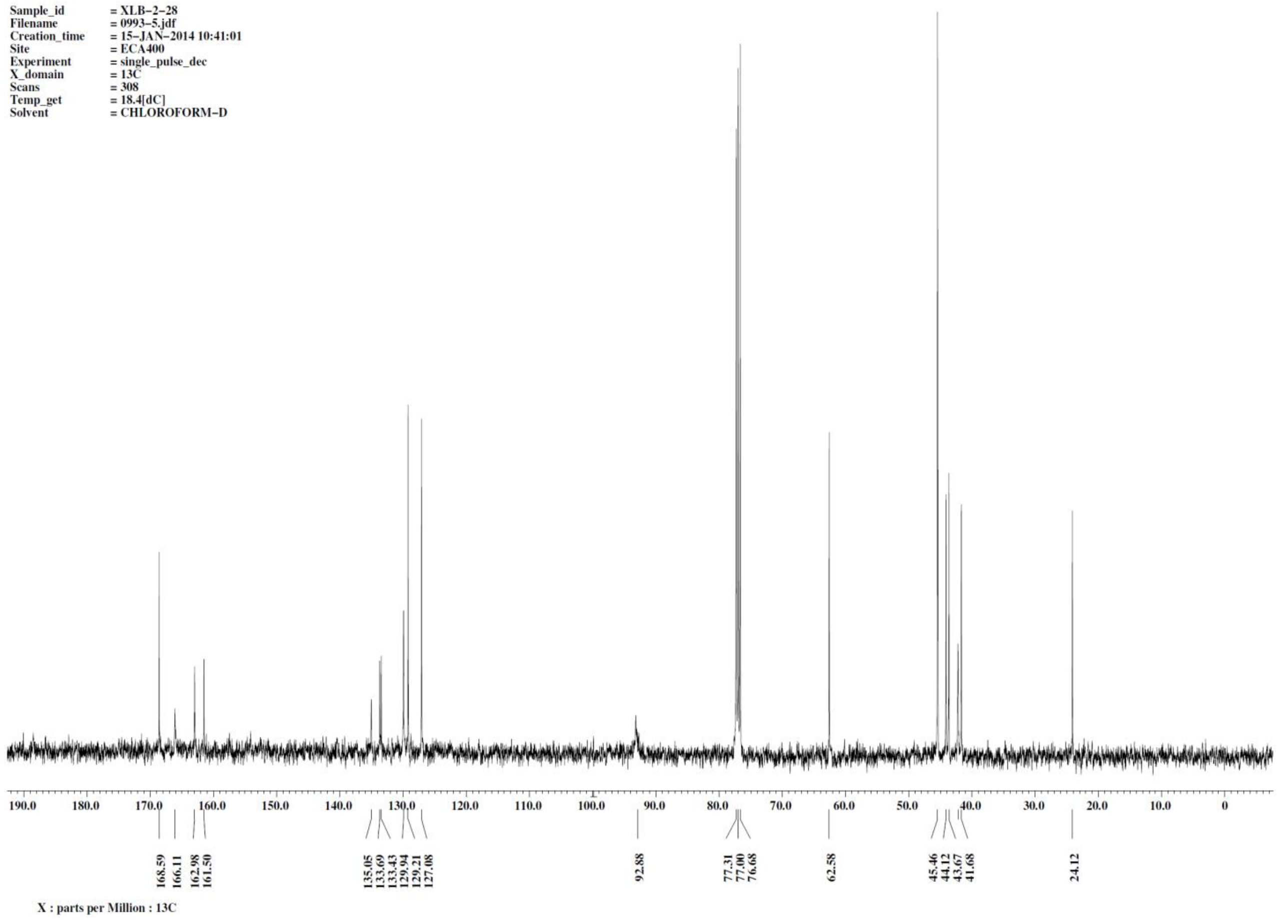

Figure S19. The ^{1}H-NMR spectrum of **12c**.

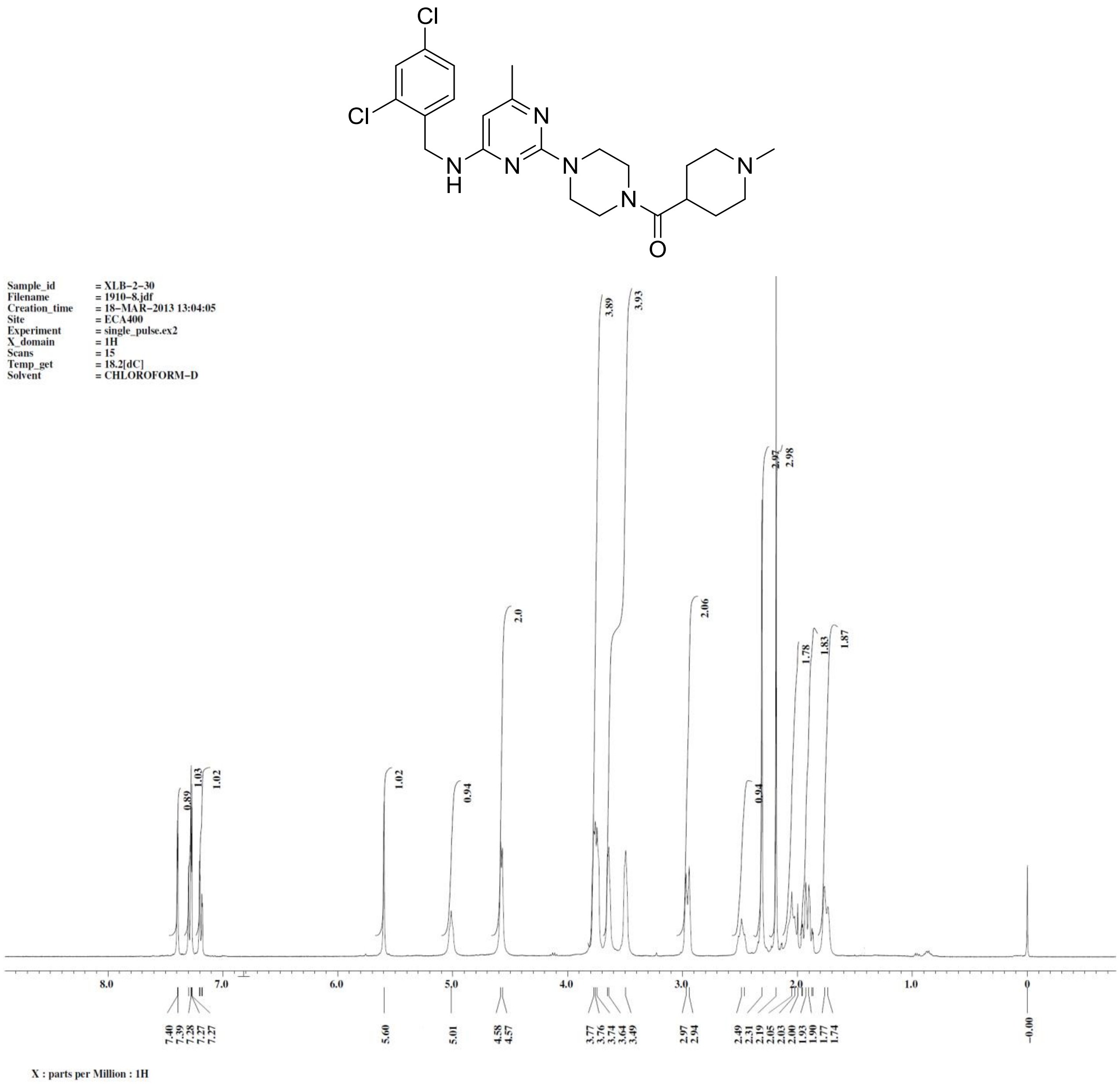

Figure S20. The ^{13}C-NMR spectrum of **12c**.

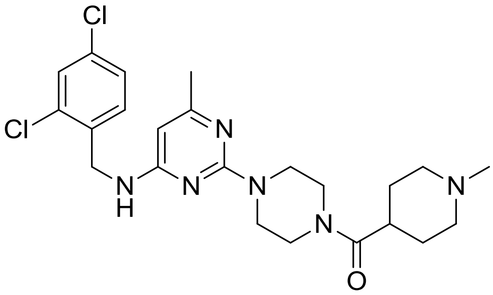

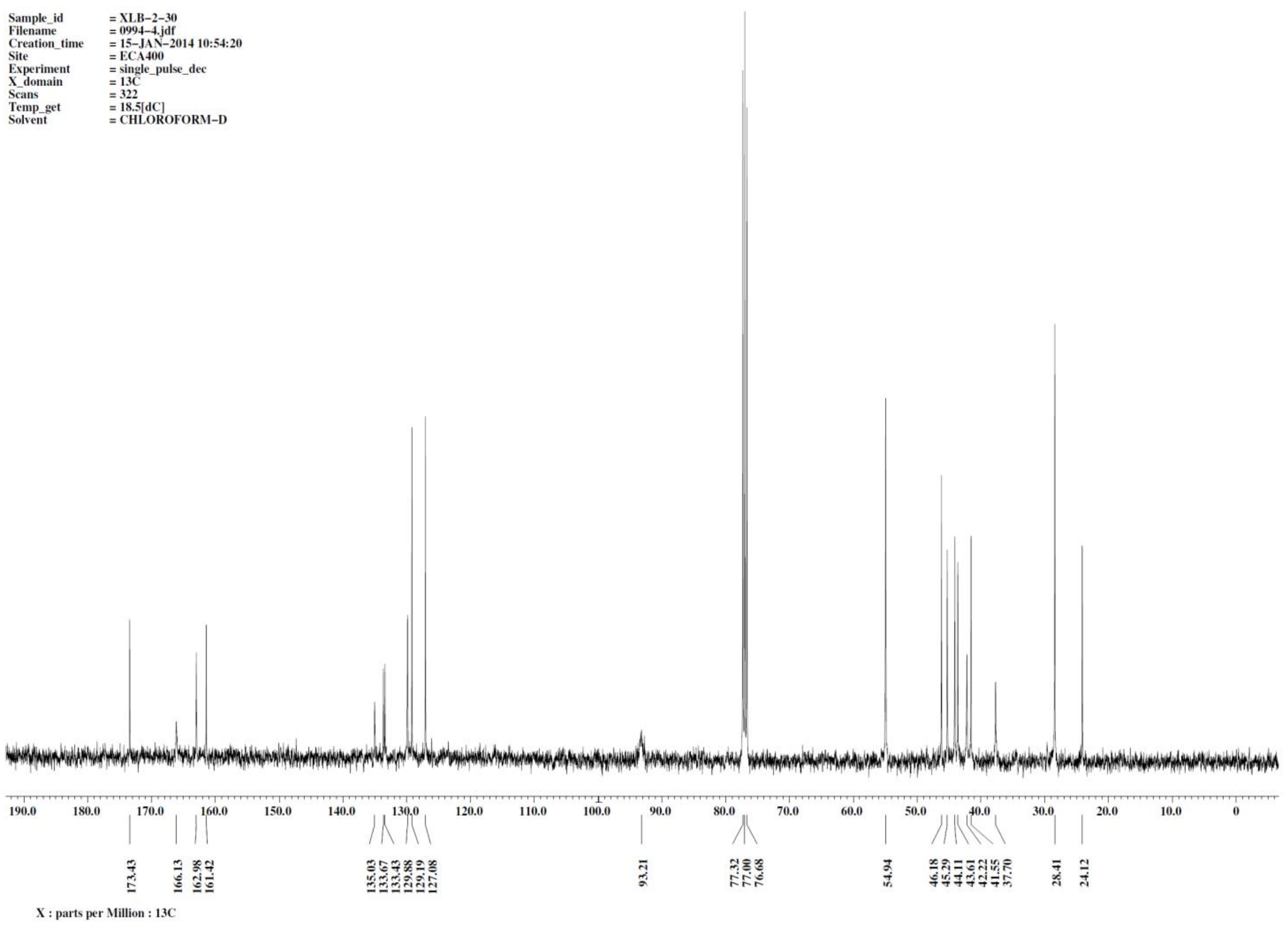

Figure S21. The ^{1}H-NMR spectrum of **12d**.

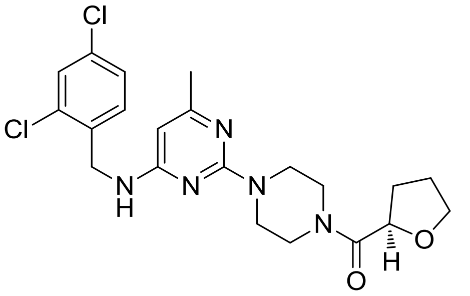

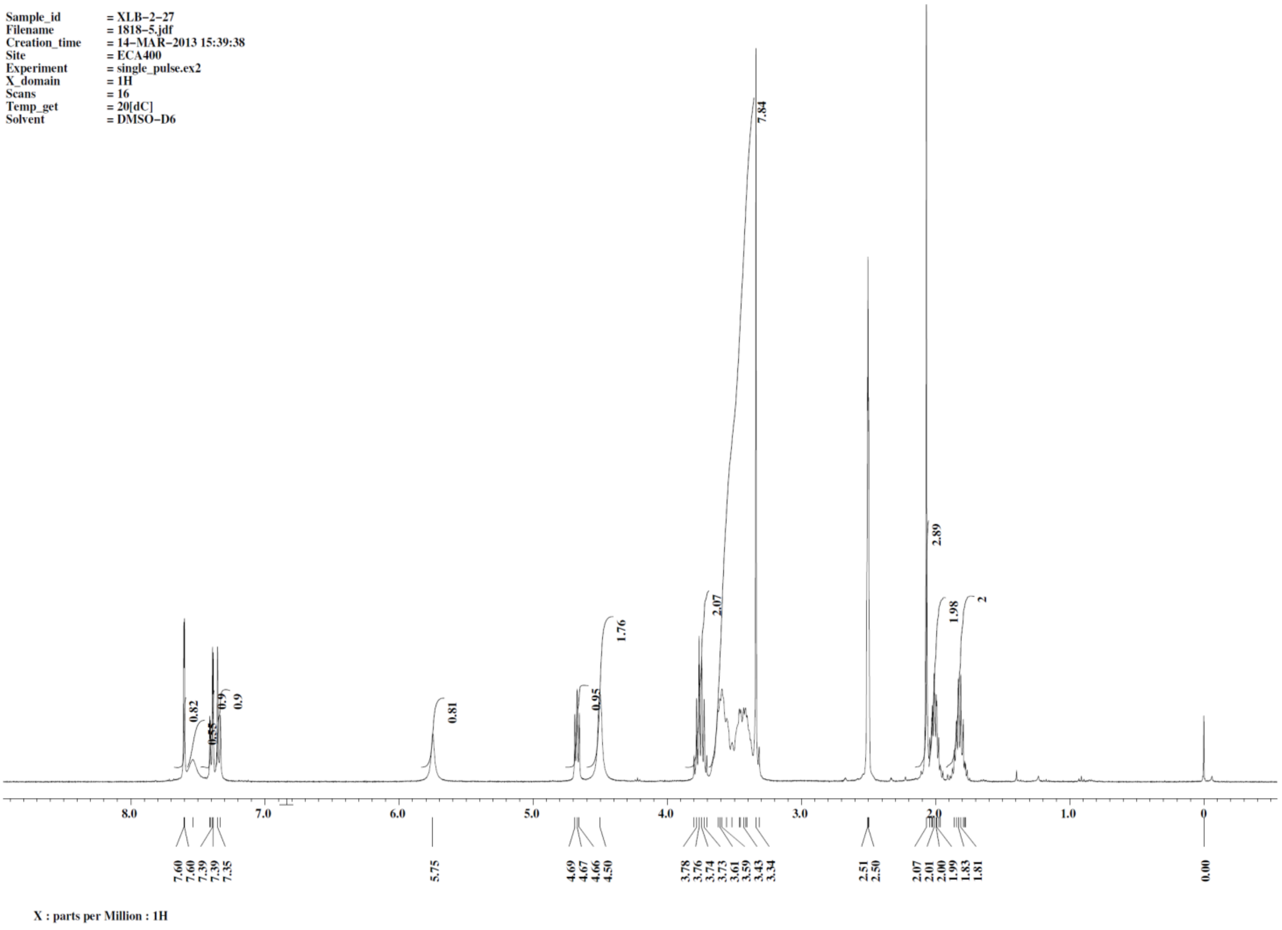

Figure S22. The ^{13}C-NMR spectrum of **12d**.

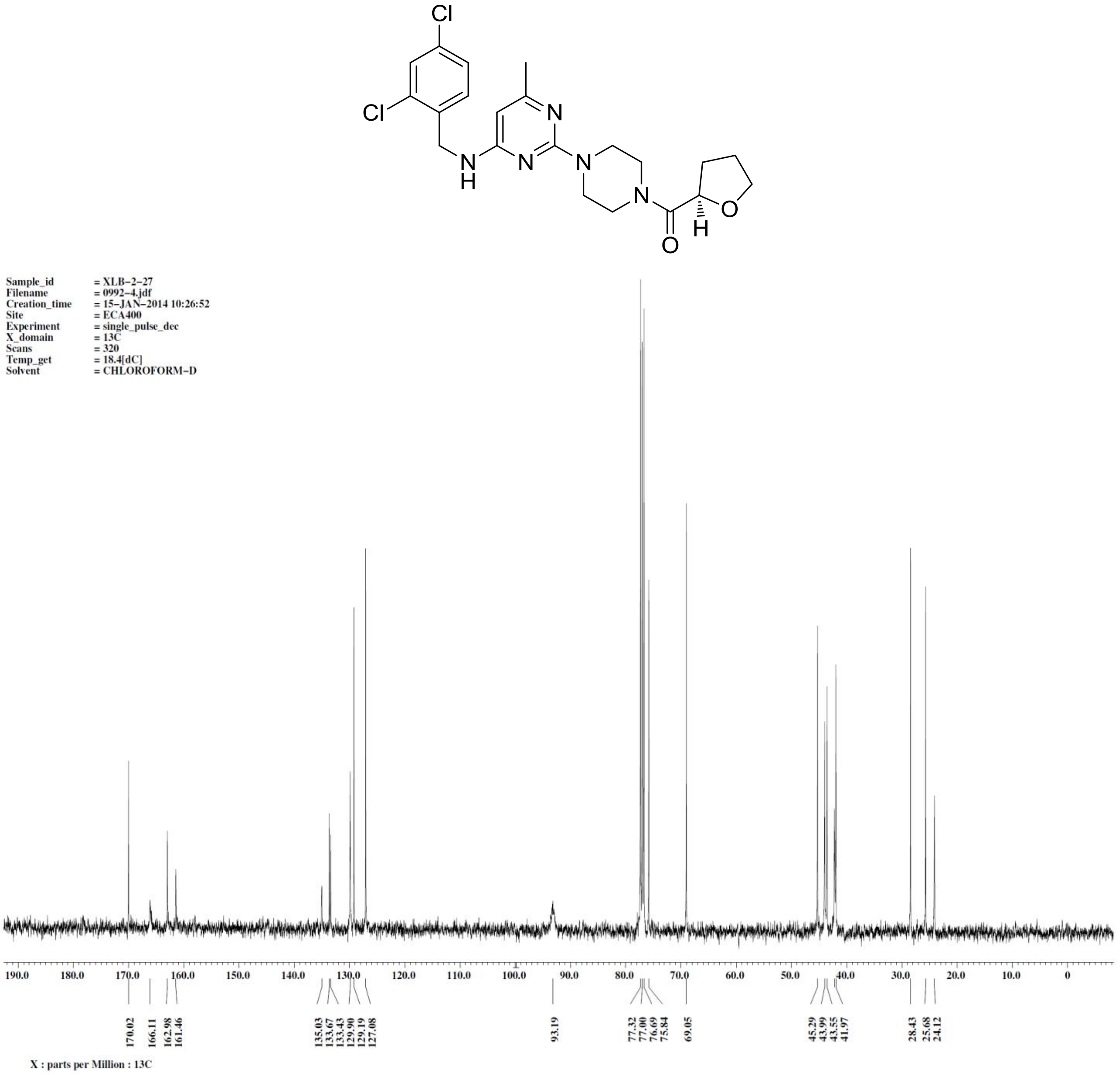

Supplement: Supplementary file 1 [file molecules-19-03539-s001.pdf]
